# Mechanical release of homogenous proteins from supramolecular gels

**DOI:** 10.1038/s41586-024-07580-0

**Published:** 2024-07-17

**Authors:** Simona Bianco, Muhammad Hasan, Ashfaq Ahmad, Sarah-Jane Richards, Bart Dietrich, Matthew Wallace, Qiao Tang, Andrew J. Smith, Matthew I. Gibson, Dave J. Adams

**Affiliations:** 1 Department of Chemistry, University of Glasgow, Glasgow, UK; 2 Division of Biomedical Sciences, Warwick Medical School, University of Warwick, Coventry, UK; 3 Department of Chemistry, University of Warwick, Coventry, UK; 4 Department of Chemistry, University of Manchester, Manchester, UK; 5 School of Pharmacy, University of East Anglia, Norwich Research Park, Norwich, UK; 6 Diamond Light Source Ltd, Diamond House, Harwell Science and Innovation Campus, Didcot, UK; 7 Manchester Institute of Biotechnology, University of Manchester, Manchester, UK

**Keywords:** Materials science, Biomaterials

## Abstract

A long-standing challenge is how to formulate proteins and vaccines to retain function during storage and transport and to remove the burdens of cold-chain management. Any solution must be practical to use, with the protein being released or applied using clinically relevant triggers. Advanced biologic therapies are distributed cold, using substantial energy, limiting equitable distribution in low-resource countries and placing responsibility on the user for correct storage and handling. Cold-chain management is the best solution at present for protein transport but requires substantial infrastructure and energy. For example, in research laboratories, a single freezer at −80 °C consumes as much energy per day as a small household^1^. Of biological (protein or cell) therapies and all vaccines, 75% require cold-chain management; the cost of cold-chain management in clinical trials has increased by about 20% since 2015, reflecting this complexity. Bespoke formulations and excipients are now required, with trehalose^2^, sucrose or polymers^3^ widely used, which stabilize proteins by replacing surface water molecules and thereby make denaturation thermodynamically less likely; this has enabled both freeze-dried proteins and frozen proteins. For example, the human papilloma virus vaccine requires aluminium salt adjuvants to function, but these render it unstable against freeze–thaw^4^, leading to a very complex and expensive supply chain. Other ideas involve ensilication^5^ and chemical modification of proteins^6^. In short, protein stabilization is a challenge with no universal solution^7,8^. Here we designed a stiff hydrogel that stabilizes proteins against thermal denaturation even at 50 °C, and that can, unlike present technologies, deliver pure, excipient-free protein by mechanically releasing it from a syringe. Macromolecules can be loaded at up to 10 wt% without affecting the mechanism of release. This unique stabilization and excipient-free release synergy offers a practical, scalable and versatile solution to enable the low-cost, cold-chain-free and equitable delivery of therapies worldwide.

## Abstract (web summary)

A stiff hydrogel gel is presented that encapsulates and stabilizes proteins without additives or excipients and uses mechanical strain to release them, offering low-cost and versatile delivery of therapies.

## Main

Consideration of the pathways of thermal protein deactivation shows that irreversible aggregation, rather than chemical degradation or unfolding, is the primary mechanism for loss of activity. For example, simple mechanical stimulation (shaking) of insulin leads to aggregation into amyloid-type fibres and loss of efficacy and bioavailability, and the gene therapy Zolgensma has just a 14-day shelf life, cannot be agitated and must be retained at 2–8 °C. Clearly, this is a barrier to wider use. Aggregation of recombinant human interferon beta leads to immunogenicity for patients with multiple sclerosis^9^. Emerging antibody-based therapies have a problem with aggregation at all stages of their life cycle, necessitating complex formulation processes.

Others have developed synthetic polymers with trehalose side-chains for protein stabilization, enabling both freeze-drying and heating stabilization, but the conjugation reduces activity^10^. However, for many therapeutic applications, covalent conjugation of a polymer using multistep synthetic chemistry and purification is not practical and has the requirement to seek regulatory approvals for each material, substantially increasing the barrier to clinical translation. This is a broad issue that means any stored protein is eventually delivered mixed with its stabilization agents, rather than the desirable solution of protein in buffer. Fully reversible hydrogels based on polyethylene glycol (PEG) have emerged to protect diverse proteins against thermal stress^11,12^ and messenger RNA formulations have been stabilized in a dissolvable matrix^13^. Similarly, frozen formulations to prevent ice crystal growth suppress protein aggregation^14^. However, in each case these gels are based on new chemical entities (and hence a substantial barrier to human use), may require a chemical stimulus (pH, sugars) to release the protein, which in practice must be dosed by the user, and take more than 1 h to release the protein. While potent, these strategies result in protein mixed with gel-forming components, which require separate approval and rigorous evaluation of their safety profile before being suitable for human use. Even materials based on PEG have immunological concerns for frequent exposure^11,12,15^.

An ideal biologic storage and transport solution would (1) remove or reduce cold-chain requirements; (2) be broadly applicable; (3) have zero or minimal excipients in the delivered protein solution; (4) not require complex chemical triggers to release; and (5) tolerate high loadings up to 100 mg ml^−1^. Here we solve each of these points using a simple, low-resource strategy. We show that low-molecular-weight supramolecular gel networks can physically entrap proteins, thereby preventing irreversible aggregation, and hence retain function at temperatures as high as 50 °C for at least 4 weeks. Upon applying pressure to push the gelated solution through a syringe filter, pure non-aggregated homogeneous and functional protein is released with all excipients trapped in the filter, ensuring only protein and buffer are delivered. This differs fundamentally from previous approaches^11^ which require a chemical trigger or dilution^16^ and do not therefore provide pure protein.

Gels can be formed by the self-assembly of low-molecular-weight gelators (LMWG)^17,18^. These are small molecules that self-assemble to give long fibrous structures which entangle to form a three-dimensional network. These gels tend to be very stiff but break at low strain. This breaking at low strain has been widely described as a drawback of these systems^19,20^. However, here we use this perceived failing as a unique benefit to enable a mechanical trigger for homogeneous protein delivery that is not possible with conventional gels.

**Fig. 1:**
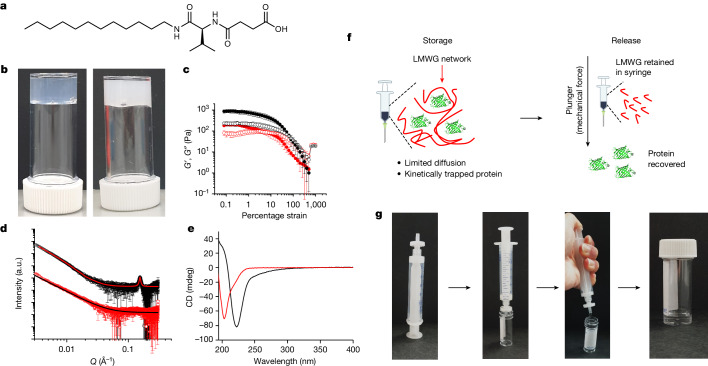
Initial gel studies. **a**, Chemical structure of the gelator used here. **b**, Photographs of example gels formed at pH 6.8 (left) and at pH 6.8 in the presence of a calcium salt (right). **c**, Typical strain sweep for gels formed in the absence (red) and presence (black) of a calcium salt, showing a high stiffness (*G*′, filled circles) but a low breakage strain. Measurements were performed in triplicates and plotted data are presented as mean ± s.d. **d**, SAXS patterns of gels made in presence of calcium chloride (black) and without CaCl_2_ (red). The fits obtained through model fitting are overlayed on each spectrum (details in Supplementary Information). The plotted data show the averaged scattering pattern obtained from five measurements across the sample. The error bars are generated during data processing by calculating scattering signal uncertainty in the detected data according to previously published methods^29,30^. **e**, Circular dichroism spectra of gels in presence of calcium chloride (black) and without CaCl_2_ (red). Circular dichroism spectra were collected in duplicates for each sample and averaged. **f**, Cartoon showing the concept of this work. **g**, Images exemplifying the syringe filter release protocol for gels. The gel is first loaded in a syringe fitted with a 0.22 μm filter. The gel is passed through the filter by gentle extrusion, releasing a clear solution. a.u., arbitrary units; CD, circular dichroism.

We formed gels from a range of LMWG (Supplementary Fig. 1). On the basis of gelation at physiological conditions, we focused on one system (Fig. 1a). The advantage of this system is that gelation can be induced by the addition of a TRIS buffer solution to a concentrated solution of the LMWG at pH 8 to give a final self-supporting material at pH 6.8. The mechanical properties can be tuned by the addition of a calcium salt (Fig. 1b). In the absence of the calcium salt, the system has relatively low storage (*G*′) and loss (*G*″) moduli, with the material breaking at low strain (less than 1%). The materials are not true gels in that there is some frequency dependence on *G*′ and *G*″, and at low frequency *G*′ and *G*″ are essentially the same (Supplementary Fig. 3e). In the presence of the calcium salt, the moduli are improved and the samples are less frequency dependent (Supplementary Fig. 3e) but, crucially, the material still breaks at low strain (Fig. 1c and Supplementary Figs. 2, 3 and 4). Using small-angle X-ray scattering (SAXS; Supplementary Fig. 5 and Supplementary Table 1) to probe the sample without salt, the scattering data can be fit simply to a power law (Fig. 1d, red), suggesting the presence of large heterogeneities in the network or irregular aggregates in the gel. The data for the gel prepared in the presence of CaCl_2_ could be fit to an elliptical cylinder model combined with a power law (Fig. 1d, black, Supplementary Fig. 6 and Supplementary Table 2). The data further present a peak at *q* = 0.154 Å (*d* = 40.8 Å), which suggests that the gelator fibres are part of a periodic arrangement in the structure with a crystalline character. Circular dichroism indicates a different arrangement in the secondary structures of the systems. The circular dichroism spectrum of the sample without any salt exhibits a negative band with a shoulder in the range 200–240 nm (Fig. 1e, red, and Supplementary Fig. 7). The sample with CaCl_2_ shows a negative band around 235 nm and a positive band occurring near 190 nm, which could be indicative of a β-sheet-like arrangement (Fig. 1e, black, and Supplementary Fig. 7).

Encapsulating additives, including proteins, in gels formed by LMWG is trivial^21,22^. The proteins are trapped in the space between the fibres; if the gel network is sufficiently permanent and the pores between fibres sufficiently small, the protein is unable to easily diffuse in the gel and hence aggregate^23^. Indeed, restricted diffusion has been shown to occur in LWMG by means of controlled release experiments^24,25^. Combining this concept with the potential for these gels to break at low strain means we can form gels within a syringe for long-term room temperature storage, followed by release through a syringe filter; the essentially insoluble extended supramolecular network will be trapped in the syringe filter, meaning that only the pure protein is released (Fig. 1f,g).

**Fig. 2:**
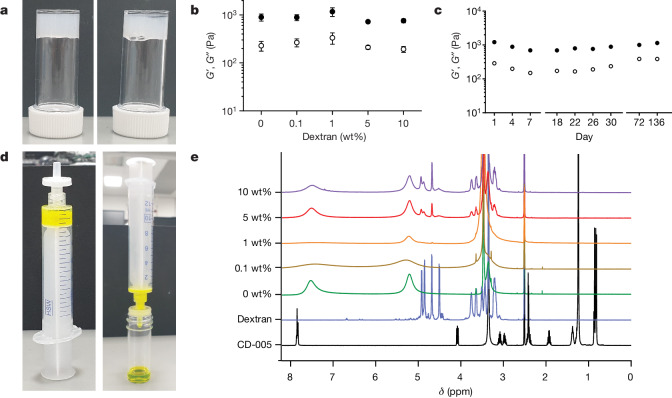
Dextran is encapsulated in, and released from, CaCl_2_-crosslinked LMWGs. **a**, Gels formed using CaCl_2_ encapsulating dextran (left, 1 wt%; right, 10 wt%). **b**, Rheological data for gels formed encapsulating dextran. Measurements were performed in triplicate and plotted data are presented as mean ± s.d. **c**, Rheological data for gels formed encapsulating 1 wt% dextran over time. Measurements were performed in triplicate and plotted data are presented as mean ± s.d. **d**, Extrusion through a syringe filter releases the entrapped material. **e**, NMR evidence showing that no gelator is released during extrusion. The peaks at around 5.0 and 7.6 ppm are from the buffer.

A model macromolecular cargo was loaded into gels to probe the effects on gel structure, function and release. Dextran (relative molecular mass *M*_r_ 6,000, hydrodynamic radius 1.6 nm)^26^ was chosen as a non-interacting additive with a similar size to insulin^27^. Dextran was added at concentrations up to 10 wt% (Fig. 2a, a value far above the loadings required for real-world drug storage and in line with recent moves towards higher-concentration biologic formulations; Supplementary Fig. 8), while keeping all other parameters constant. Our gelation approach is almost instantaneous: in comparison, chemically triggered hydrogel storage solutions require the addition of dosed release agents, such as sugars and require up to an hour to gel^11^. The gel properties were retained over all loadings, which is essential for a broadly applicable storage solution (Fig. 2b). Extended aging experiments showed that these properties did not change significantly over at least 136 days (Fig. 2c). The gels can be formed in situ inside a syringe. The added dextran does not affect the gelator’s packing (Supplementary Fig. 8 and Supplementary Table 2). As initial proof-of-concept, we entrapped a fluorescent dextran within a gel and then extruded it through a syringe filter. Pure dextran was collected directly (Fig. 2d), with 92 ± 2% of the expected amount collected, showing that there is little entrapped permanently in the network (Supplementary Fig. 9). No gelator could be detected in the extruded liquid by ^1^H NMR spectroscopy (Fig. 2e), showing that the gel network is trapped in the syringe filter and our simple approach leads to excipient-free homogeneous protein on demand. The gelator is trapped in the filter as shown by Fourier-transform infrared spectroscopy (FTIR) data (Supplementary Fig. 10). To add further quantification, we used LMWG which incorporate an inherently fluorescent group. After extrusion, very low fluorescence could be detected in the extrudates (less than 5 ppm; Supplementary Fig. 11), showing the power of this approach (Supplementary Figs. 12–16).

**Fig. 3:**
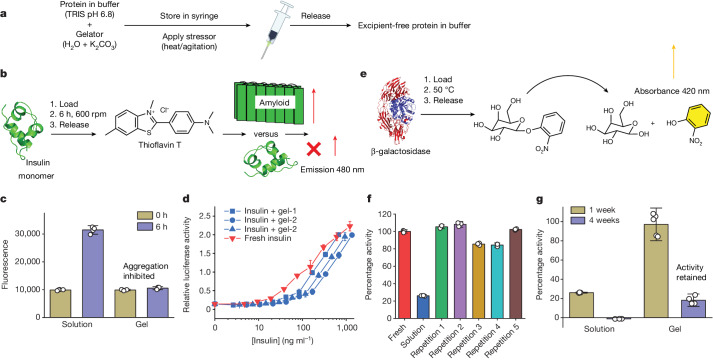
Retention of protein function in hydrogels. **a**, General preparation process for gel. **b**, Probe for aggregation inhibition using insulin with thioflavin T assay. **c**, Quantification of thioflavin T assay (*n* = 5); error bars, mean ± s.d. **d**, Cell-based assay for CD220 recognition by insulin following recovery from gel with shaking at 600 rpm for 24 h, compared to fresh (*n* = 2); error bars, mean ± s.e.m. **e**, The β-galactosidase assay for post-recovery function. **f**, Activity recovery after 7 days at 50 °C for independent repeats (*n* = 3); error bars, mean ± s.d. **g**, Comparison of 1 and 4 weeks storage and recovery of β-galactosidase (solution for 1 and 4 weeks, *n* = 3; gel for 1 week, *n* = 5; gel for 4 weeks, *n* = 4); error bars, mean ± s.d.

With confirmation that macromolecules can be encapsulated and selectively released by mechanical trigger, protein storage was undertaken to demonstrate recovery and the mode of action (preventing aggregation). As a first challenge, we chose to encapsulate (bovine) insulin as a model therapeutic protein (Fig. 3a). Insulin is one of the most widely used protein drugs in the world but must be stored cool or freeze-dried, requiring the user to self-prepare the solution before injection^28^. The instructions for insulin are very specifically not to shake, as it is prone to aggregate into extended amyloid fibres, losing efficacy and bioavailability. After initial screening using dynamic light scattering, insulin was loaded into gels (with no Ca^2+^ in this case), which were then incubated at 25 °C and shaken at 600 rpm: this is very aggressive agitation, far beyond a real-world stress. After this, pure excipient-free protein was recovered by action of the syringe and any aggregation to amyloid was determined by the thioflavin T assay (Fig. 3a). All samples were treated identically, and the solution-only samples were passed through the syringe filter, to exclude effects of the filtering process on aggregation or the possibility that the filter could exclude aggregatess. Limited aggregation was detected in the gel-loaded samples, but extensive aggregation occurred in the absence of the gel. Saturation transfer difference (STD) nuclear magnetic resonance (NMR) spectroscopy strongly implies that that the insulin is not adsorbed onto the fibres of the gel while encapsulated (Supplementary Fig. 17). The rheological properties of the gels are somewhat affected by the addition of insulin (Supplementary Fig. 18), but the overall process is still effective; in no case was gelator detected in the solutions extruded through the syringe filter (Supplementary Figs. 19 and 20), although overlapping of peaks makes the analysis more difficult than in the dextran case. Complete recovery of the expected volume of insulin (100% taking into account the volume of the syringe filter used to collect the gelator) was achieved in the extruded sample (Supplementary Fig. 21). Mass spectrometry before and after the encapsulation and release demonstrate the chemical stability of the insulin under these conditions (Supplementary Fig. 22). Additionally, SAXS shows that the scattering from a solution of insulin before encapsulation and from a solution after encapsulation and release is very similar, demonstrating a lack of aggregation (Supplementary Fig. 23). Hence, our approach provides dual protection against aggregation and allows protein release in a pure form. Indeed, we can encapsulate insulin at a concentration of 3.2 mg ml^−1^, a concentration around that of commercial formulations (U100), with complete release of excipient-free insulin (Supplementary Figs. 19 and 20). We also tested two structurally related LMWG (Supplementary Fig. 24), which protected insulin against aggregation, confirming that our mode of action is linked to physical immobilization, not a specific interaction. Finally, a cell-based assay confirmed that released insulin retained CD220 binding, equal to fresh, demonstrating that it is correct folded and retains biological activity (Fig. 3d).

As a further test, β-galactosidase was encapsulated (with added Ca^2+^). The β-galactosidase catalyses the decomposition of *o*-nitrophenyl-galactoside allowing colorimetric evaluation of activity. The gels were placed in an incubator at 50 °C (a stringent test beyond feasible transport conditions) for 7 days (Fig. 3e). The rheological properties of samples at this temperature are still gel-like as long as the calcium salt is present (Supplementary Fig. 25), but calcium does reduce the recovered protein yield. After this time, protein was recovered by simple syringe action. Compared to fresh, β-galactosidase in buffer alone retained just 26.1 ± 0.4% of its function but across 15 repeats (3 technical, 5 biological) the gel-stored protein recovered 84.4–108.1% of function (per protein mass), with a mean of 97.1 ± 2.8% (Fig. 3f). This is equal performance to a multi-armed boronic acid function PEG hydrogel^11^ but our technology functions at biomedically relevant volumes (0.5 ml versus 50 μl), does not require addition of any triggers, such as sugars or acid, and the resulting protein was excipient-free. As an even more challenging test, gels were stored at 50 °C but for 4 weeks. This removed all activity from the solution-stored β-galactosidase but the gel-stored protein retained up to 20% of its function (Fig. 3g): this is a remarkable level of recovery from a harsh storage condition at conditions that even the hottest locations on earth rarely face. To show that the gels are robust, samples were mailed through the UK postal service to experience a realistic distribution challenge (shaking, dropping, temperature range; Supplementary Fig. 26). Over the 2 day delivery, the gels were recovered intact and about 100% activity was retained, showing that this method could conceptually be used for the postal delivery of active proteins for healthcare (Supplementary Fig. 27).

We have demonstrated that protein function is retained in stiff low-molecular-weight gels when stored at temperatures as high as 50 °C for up to 4 weeks by preventing irreversible aggregation. Owing to the unique mechanical properties of the low-molecular-weight gels, they break when passed through an in-line syringe filter, thereby releasing the fully functional protein cargo with no need for any chemical triggers, and the effect is instantaneous. Owing to the extended supramolecular network formed by the gelators, all the gel components (for example, excipients) are retained in the filter, meaning that only protein and buffer are released. This means only pure protein in buffer is delivered, unlike all competing technologies which lead to protein containing new, often untested, excipients which would then be delivered to a patient and hence face regulatory and safety barriers to use. For example, others have described a system encapsulating a therapeutic to maintain function. In their case, release occurs on dilution, meaning that the polymer gel components are still present^16^. Similarly, encapsulation and stability have been shown in a multiarm PEG gel^11^. A release solution is required to break the gel, meaning that delivery would contain the gel components and release solution. Hence, our excipient-free approach is not just an iteration but a major step forward. This is also a benefit over freeze-dried or frozen formulations, as it requires neither on-use reconstitution nor energy-intensive ultra-low freezers, respectively. We have therefore provided a new scalable means of storing, transporting and safely using biologics, affording a simple and effective strategy to deliver biologic therapies to low-resource settings for which they are needed most.

## Methods

All chemicals were purchased from Sigma-Aldrich and used as received. Deionized water was used throughout. The gelator used here was synthesized following the protocols of ref. ^31^. Full details are provided in the Supplementary Information (p. 25). The other gelators tested (Supplementary Fig. 1) were prepared as described elsewhere^32^.

### pH measurements

The pH of solutions and gels was measured using an FC200 pH probe (Hanna instruments) calibrated using pH 4.01, 7.01 and 10.01 buffer solutions. The probe was rinsed with deionized water between measurements.

### Rheology

Rheological measurements were carried out using Anton Paar Physica MCR301 and M101 Rheometers. A cup and vane (ST10-4V-8.8/97.5-SN42404) system was used for all frequency and strain sweeps, with a measuring gap of 1.35 mm. Gels were prepared directly in 7 ml Sterilin vials, which were loaded on to the rheometer and measured in situ to ensure that no damage was carried out to the gels by transfer from vials. Strain sweeps were performed from 0.08% to 1,000% at a frequency of 10 rad s^−1^. Frequency sweeps were performed from 1 to 100 rad s^−1^ at a constant strain of 0.1% (within the linear viscoelastic region for all gels). To test the behaviour of the materials at low frequency, frequency sweeps were further collected from 0.01 to 100 rad s^−1^ at a constant strain of 0.1% (within the linear viscoelastic region for all gels). To measure the yield stress of the gels, strain sweeps were performed at a frequency of 1 rad s^−1^ from 0.1% and 1,000%. The yield stress value was obtained by plotting the elastic stress (*G*′ *γ*_0_) against the strain amplitude, according to previously published methods^33–37^.

To obtain frequency sweeps of the samples at higher temperatures, gels were prepared directly in 7 ml aluminium cups and loaded on to the rheometer. The temperature was raised linearly from 25 to 60 °C with a heating rate of 2 °C min^−1^. Then, frequency sweeps were collected from 1 to 100 rad s^−1^, while keeping a constant strain of 0.1% and temperature of 60 °C.

### Small-angle X-ray scattering

SAXS experiments were performed at Diamond Light Source, at the I22 beamline^38^. The beamline operates at an energy of 12.4 keV and the camera length was set to 4.275 m to give a *q* range of 0.002–0.30 Å^−1^. The gels were prepared as described in the next section and immediately loaded in glass capillaries using a 1 ml syringe with a 21 G needle. The raw data were processed using the DAWN Science software (v.2.27)^39^, according to a standard I22 pipeline^30^. As part of the processing, the backgrounds were subtracted from the raw two-dimensional SAXS data and a full azimuthal integration was performed to reduce the data to an *I* versus *q* plot. The plots were then fitted to structural models using the SasView software (v.5.0.4).

BioSAXS experiments were performed at the B21 beamline at Diamond Light Source. The beamline operates at a fixed energy of 13 keV and a camera length of 3.600 m to obtain a *q* range of 0.0031–0.38 Å^−1^.

A total 50 μl of each sample was loaded into a 96-well plate and measured using the BioSAXS EMBL Arinax sample-handling robot. For each sample, 30 × 1 s frames were collected at 20 °C. The two-dimensional raw data were processed in the DAWN Science software (v.2.27)^39^ to yield the *I* versus *q* plots. The data were then averaged and the buffer background was manually subtracted using the ScÅtter software (https://bl1231.als.lbl.gov/scatter/, R. P. Rambo).

### Formation of gels for CD-005

CD-005 gels were prepared either with or without the addition of CaCl_2_. For both, 50 mg of CD-005 was added to 20 mg of K_2_CO_3_ and dissolved in 5 ml of deionized water under overnight stirring. For the samples without CaCl_2_, 0.4 ml of the gelator solution was transferred to a vial, to which 1 ml of 1.5 M Tris HCl (pH 6.8) was added. For the samples formed with the calcium trigger, 5.5 µl of a 200 mg ml^−1^ CaCl_2_ solution was first added to 1 ml of 1.5 M Tris HCl (pH 6.8) in a separate vial. This was then added in one aliquot to 0.4 ml of gelator buffer. For rheological measurements, the gels were prepared in vials and left undisturbed overnight on the bench. To prepare the samples in syringes, the gels were prepared as above and immediately transferred (less than 1 min) to a syringe through a 21 G needle. All the samples needed to be prepared at 19 °C to ensure optimal gelation.

### Preparation of other tested gels by reduction in pH using GdL

Micellar solutions were prepared as previously described at concentrations of 5 mg ml^−1^ (ref. ^32^). Gel samples were prepared in 7 ml Sterilin vials by addition of 2 ml of stock solution (adjusted to pH 10.5) to 16 mg of solid glucono-δ-lactone (GdL) for 5 mg ml^−1^ of solutions. The vials were swirled briefly by hand to ensure complete dissolution of GdL then left to stand overnight undisturbed.

### Encapsulation of dextran, insulin and β-galactosidase

For the CD-005 gels, dextran was encapsulated in the gel by dissolution of dextran powder in 1.5 M Tris HCl, pH 6.8 at a range of concentrations (0.14–14 wt%). A total 1 ml of this solution was transferred to 0.4 ml of the gelator solution described above to achieve the desired final concentrations of dextran (0.1–10 wt%). For the gels obtained by reduction in pH, the dextran was dissolved at the various required concentrations in deionized H_2_O under stirring. These solutions were then used to prepare the micellar solutions of the gelators at 5 mg ml^−1^.

### Release protocols through syringe filter

To release the gel with dextran inclusion, the gel prepared in the syringe was passed through a 2.7 μm filter. Figure 1g exemplifies the methodology of release through the filter: the sample is first allowed to gel overnight in a syringe, then the gel is gently passed through the 2.7 μm syringe filter, which releases a clear solution. Generally, about 80% of the liquid is recovered from the procedure.

### Release protocols for UV–Vis and fluorescence

For the quantification of released insulin and dextran, samples were prepared as follows. Insulin was dissolved in 1.5 M Tris HCl (pH 6.8) at concentrations of 0.28 and 4.48 mg ml^−1^. To avoid aggregation, the samples were left overnight on a roller at 76 rpm to ensure complete dissolution. To form gels in the syringes, 0.4 ml of the CD-005 gelator solution prepared as described above was transferred to a 12 ml syringe. Then, 1 ml of the insulin solution in Tris was transferred using a 5 ml syringe and a 21 G needle directly in the 12 ml syringe. This resulted in quick formation of the gels at final concentrations of 0.2 and 3.2 mg ml^−1^. The gels were left overnight to stabilize and then gently passed through a 2.7 μm filter, releasing a clear solution. Ultraviolet–visible (UV–Vis) light was measured directly on the obtained solution. To quantify the amount of insulin released, insulin solutions were prepared at a range of concentrations (0.2–4.48 mg ml^−1^) in a similar way in 1.5 M Tris HCl.

For the quantification of released dextran, 1.4 wt% (14 mg ml^−1^) solution of fluorescein isothiocyanate dextran was prepared in 1.5 M Tris HCl buffer by dissolving 14 mg of the dextran in 1 ml of buffer. A total of 5.5 μl of 200 mg ml^−1^ of CaCl_2_ solution was then added to 1 ml of this solution and swirled briefly. To form gels in syringes, a similar method was used as described above, achieving a final concentration of 1 wt% (10 mg ml^−1^). The gels were left overnight to stabilize and then gently passed through a 2.7 μm filter. The resulting dextran solution was too concentrated to study by means of UV–Vis and fluorescence. For UV–Vis, a dilution of a factor of 10 was carried out using buffer, reaching a final concentration of 1 mg ml^−1^. For fluorescence, a factor of 100 was needed (0.1 mg ml^−1^) to avoid self-quenching at higher concentrations. To quantify the amount of released dextran, fluorescein isothiocyanate dextran was dissolved in 1.5 M Tris HCl buffer at desired concentrations (1–0.05 mg ml^−1^), ensuring the same pH throughout.

### Circular dichroism

Circular dichroism data were acquired on a Chirascan VX spectrometer (Applied Photophysics) using a quartz cuvette with a 0.01 mm path length. The spectra were collected in the range 180–400 nm with a scanning step size of 1.0 nm and scanning rate of 0.25 s at room temperature. The samples were prepared in Sterilin vials as described, keeping the same volumes of the components. Small amounts of the gels were then transferred to the cuvette before measurement.

### FTIR spectroscopy

Data were recorded using an Agilent Cary 630 FTIR spectrometer (with ATR attachment). The filter paper from the 2.7 μm filter was removed by carefully opening the syringe filters. Then, the background of the empty ATR crystal was taken. Small amounts of a clean filter and one after extrusion were deposited on the ATR crystal to record the spectra.

### UV–Vis

Absorption spectra were recorded on an Agilent Cary 60 UV–Vis spectrophotometer using a quartz cuvette with 0.1 mm path length. Samples were prepared as above and 300 μl of the solution was transferred to the cuvette using a 200 μl pipette.

### Fluorescence

Fluorescence data were collected using an Agilent Technologies Cary Eclipse fluorescence spectrometer. Samples were prepared as described above at a 2 ml volume and transferred to a quartz cuvette with a 1 cm path length. For the fluorescein isothiocyanate dextran, the excitation wavelength was 470 nm. For the 2NapIF release studies, the excitation length was 320 nm. In all cases, the excitation and emission slit widths were 5 nm and 5 nm. To quantify the amount of 2NapIF in the extrudate, a known volume of the extruded sample was freeze-dried (1 ml) and then fully redissolved in DMSO.

### NMR spectroscopy

Water suppression and STD experiments (Supplementary Fig. 17) were recorded on a Bruker spectrometer operating at 499.31 MHz and equipped with a Neo console and Bruker 5 mm SmartProbe. The ^1^H experiments were recorded using the perfect echo WATERGATE sequence of ref. ^40^ incorporating the double echo W5 sequence of ref. ^41^. The delay between successive pulses in the selective pulse train was set at 333 μs, corresponding to 3,000 Hz between the null points. The ^1^H spectra were acquired in four dummy scans and 128 scans with a relaxation delay of 1 s and signal acquisition time of 4.2 s. STD spectra were obtained using the same sequence but with an overall relaxation delay of 5 s. Presaturation was applied during the final 4 s of the relaxation delay using a train of 100 Gaussian pulses (40 ms) with peak powers of 243 Hz at 100 ppm (off resonance) and −3.8 ppm (on resonance) in separate experiments, which were recorded with 16 dummy scans and 16 scans. Spectra were processed with an exponential line broadening factor of 1 Hz and referenced to the CH_3_ triplet of ethanol (1.2 ppm) present as an impurity in our commercial insulin sample^42^.

The 3.2 mg ml^−1^ of insulin solution and the CD-005 gel were prepared and aged in 5 mm NMR tubes (Wilmad 528-PP) for 20 h at 22 °C.

### High-resolution mass spectrometry

High-resolution mass spectrometry was recorded using a ThermoScientific Exactive Plus Orbi-Trap with ESI ionization at the University of Strathclyde, Glasgow. For the analysis, the sample is directly injected by means of the UHPLC of 10 μl of sample into the solvent flow (0.1% formic acid in methanol). The sample is injected for 1.8 min at a flow rate of 0.1 ml min^−1^. The mass spectrometer uses positive/negative polarity switching to obtain both the positive and negative mass spectrum co-currently, with a scan range of 400–6,000 Da at a resolution of 70,000. On the basis of the predicted mass, the interest molecular peak is mass matched in the spectrum with 4 decimal point accuracy (isotopic discrimination levels). For the insulin-containing samples, the mass was best detected at 3+ charge.

### Insulin-loaded gel preparations

#### NapIF

First, 1.1 ml of 0.1 M NaOH was added to 8.9 ml of deionized H_2_O. A total of 50 mg of NapIF was then added to this solution and left to dissolve overnight on a rotary shaker. Before gelation, insulin is added to a final concentration of 0.2 mg ml^−1^. Gelation is initiated by adding 80 mg of GdL to this solution. The sample is then pipetted into 5 × 2 ml syringes before being sealed with parafilm and left overnight.

#### NapFF

First, 1 ml of 0.1 M NaOH was added to 9 ml of deionized H_2_O. A total of 50 mg of NapFF was then added to this solution and left to dissolve overnight on a rotary shaker. Before gelation, insulin is added to a final concentration of 0.2 mg ml^−1^. Gelation is initiated by adding 20 mg of GdL to this solution. The sample is then pipetted into 5 × 2 ml syringes before being sealed with parafilm and left overnight.

#### CD-005

A total of 20 mg of potassium carbonate was dissolved in 5 ml of deionized H_2_O. A total of 50 mg of CD-005 was added to this solution and left to dissolve overnight on a rotary shaker. A total of 4 ml of this solution was added to 10 ml of 1.5 M Tris HCl at pH 6.8. Immediately after, insulin was added to a final concentration of 0.2 mg ml^−1^. The sample was then pipetted into 5 × 2 ml syringes before being sealed with parafilm and left overnight.

### Insulin storage and assay

Insulin in solution, along with insulin in gel (in syringe), were agitated on an Eppendorf Smartblock at 600 rpm, 25 °C for 6 h. Before analysis, the samples in syringes was passed through a 0.22 μm filter to separate protein from the gelators and then used in the assays.

### Dynamic light scattering

Dynamic light scattering was used to measure the hydrodynamic radius on a Zetasizer ZS (Malvern Panalytical). Measurements were carried out using a 4 mW He-Ne 633 nm laser module operating at 25 °C at an angle of 173° (back scattering) and results were analysed using Malvern DTS 7.03 software. There were ten replications for each of the samples with at least 12 measurements recorded for each run.

### Thioflavin T assay

A 1 mM stock solution of thioflavin T was prepared in H_2_O. This thioflavin T was diluted in PBS (pH 7.4) so that the final thioflavin T concentration in each well was 25 μM in 100 μl. Another 100 μl of insulin solution from each of the gelators after passing through 0.22 μm filter was added to the wells. Thioflavin T fluorescence was measured using a fluorescence microplate reader (excitation 450 nm, emission 485 nm).

### Cell-based assay for insulin function

Insulin in solution and in gel were prepared as described above. In brief, 50 mg of CD-005 (gelator) was dissolved in 5 ml of K_2_CO_3_ (20 mg) solution overnight the day before experiment. Insulin was dissolved in 1.5 M Tris HCl buffer (pH 6.8) at protein concentration of 0.2 mg ml^−1^ and then added into 0.4 ml of gelator solution. The mixture was immediately transferred into a 3 ml syringe. All the syringes, including insulin solution only, gelator solution only, Tris HCl buffer only and insulin in gel, were agitated on Eppendorf Smartblock at 600 rpm, 25 °C for 6 h. Before insulin activity cell assay, all the samples were passed through a 0.22 µm filter to separate protein from gelator.

iLite Insulin Assay Ready Cells (purchased from Svar Life Sciences) was used as received to test insulin activity and assay was performed according to manufacturer’s instructions. Cells were quickly thawed in 37 °C water bath, 250 µl of which was diluted to 6 ml using full culture media (RPMI supplied with 10% FBS and 1% PSA). A total of 40 µl of cell diluent was then mixed with insulin solution at equal volume and incubated in white tissue culture plate at 37 °C for a further 5 h. Firefly luciferase substrate of 80 µl was then added into cells and incubated at room temperature for 15 min. The whole plate was read on plate reader for firefly luciferase luminescence intensity. Specifically, fresh insulin solution was prepared to make a calibration curve with insulin stock concentration ranging from 2,000 to 0 ng ml^−1^, where 1,000 ng ml^−1^ was selected as insulin activity test concentration. Concentration of separated insulin sample was determined by A280 on NanoDrop and calculated on the basis of protein sequence. Protein was diluted to indicated concentrations for activity test. Dilution of gel only and buffer only was identical to protein dilution.

### β-galactosidase storage and assay

Stock solutions of the gelator and protein were freshly prepared before mixing. A total of 20 mg of KCO_3_ was dissolved in 5 ml of distilled water, to which 50 mg of CD-005 (gelator) was added. This mixture was stirred overnight to dissolution. In a separate vial, 5.5 µl of 200 mg ml^−1^ of CaCl_2_ solution was added to 1 ml of 1.5 M Tris HCl, pH 6.8. The β-galactosidase was added to give a 10 mg ml^−1^ solution. A total of 0.4 ml of the gelator in buffer was transfered to a vial, to which 1 ml of the protein solution was added. These were agitated and rapidly (less than 1 min) transferred into the syringe.

Gel-loaded syringes were stored in a thermostated incubator for the indicated amount of time, before being retrieved and assayed for function. Protein was recovered from the syringe by passing through a syringe filter (0.22 mm). A total of 50 µl of 100 µg ml^−1^ of β-galactosidase solution (this was adjusted using PBS as TRIS can inhibit the activity of this protein) was added into the wells of a 96-well plate, containing 100 µl of 16 mM oNPG (4.82 mg ml^−1^). The absorbance was then measured at 420 nm each minute for 10 min. Activity was normalized to total protein mass using a standard BCA assay, as the non-optimized calcium stabilized gels did not give 100% recovery from a single extrusion.

### Postage protocols

Gels containing β-galactosidase were prepared as described above. Five gels and one solution of β-galactosidase (that is, in the absence of gel) were triple-bagged and put in a parcel. They were posted using Royal Mail signed delivery. A temperature logger was included in the parcel. In total, the package was in delivery for 3 days. A readout of the temperature logger is shown in Supplementary Fig. 12 (the range was 17.6–24.2 °C). On receipt, samples were visually inspected for leaks (none seen) and to confirm that the gels were intact. Samples were recovered and assayed as above.

### Reporting summary

Further information on research design is available in the Nature Portfolio Reporting Summary linked to this article.

## Online content

Any methods, additional references, Nature Portfolio reporting summaries, source data, extended data, supplementary information, acknowledgements, peer review information; details of author contributions and competing interests; and statements of data and code availability are available at 10.1038/s41586-024-07580-0.

### Supplementary information

Supplementary Information

Reporting Summary

## Data Availability

All data are available in the main text or the Supplementary Information or available on reasonable request.
