# Supplementary material for: Mechanical release of homogenous proteins from supramolecular gels
Source: Nature. 2024 Jul 17;631(8021):544–8. doi: 10.1038/s41586-024-07580-0 (PMC11254749; doi:10.1038/s41586-024-07580-0)
Supplement: Supplementary file 1 — Supplementary Information [file 41586_2024_7580_MOESM1_ESM.pdf]

nature portfolio

https://doi.org/10.1038/s41586-024-07580-0

Supplementary information

Mechanical release of homogenous proteins from supramolecular gels

In the format provided by the authors and unedited

Supplementary Information

Additional data

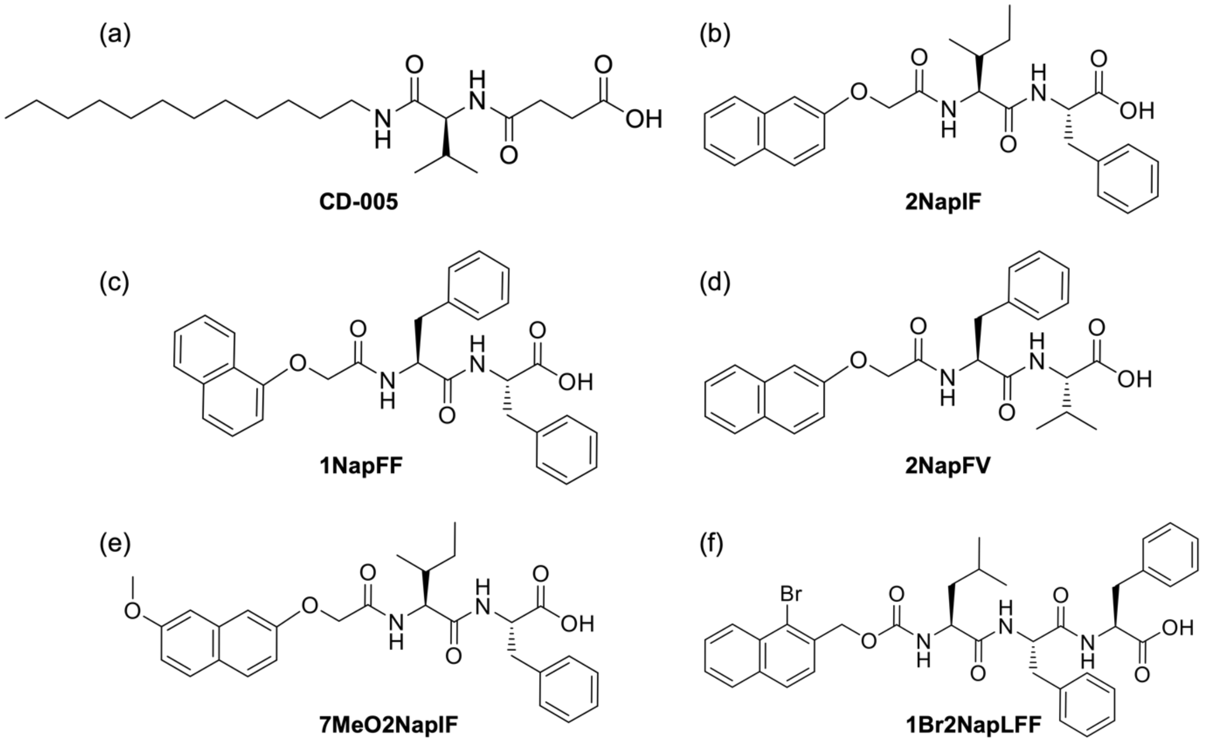

Figure S1. Example gelators used for our encapsulation and release method. For (a), the gelator we focus on in this work, gelation was achieved by adding a concentrated solution at pH 8 to a buffer. For (b)-(f), gels were formed by a pH switch from a solution at pH 10.5 giving gels at a final pH 4.

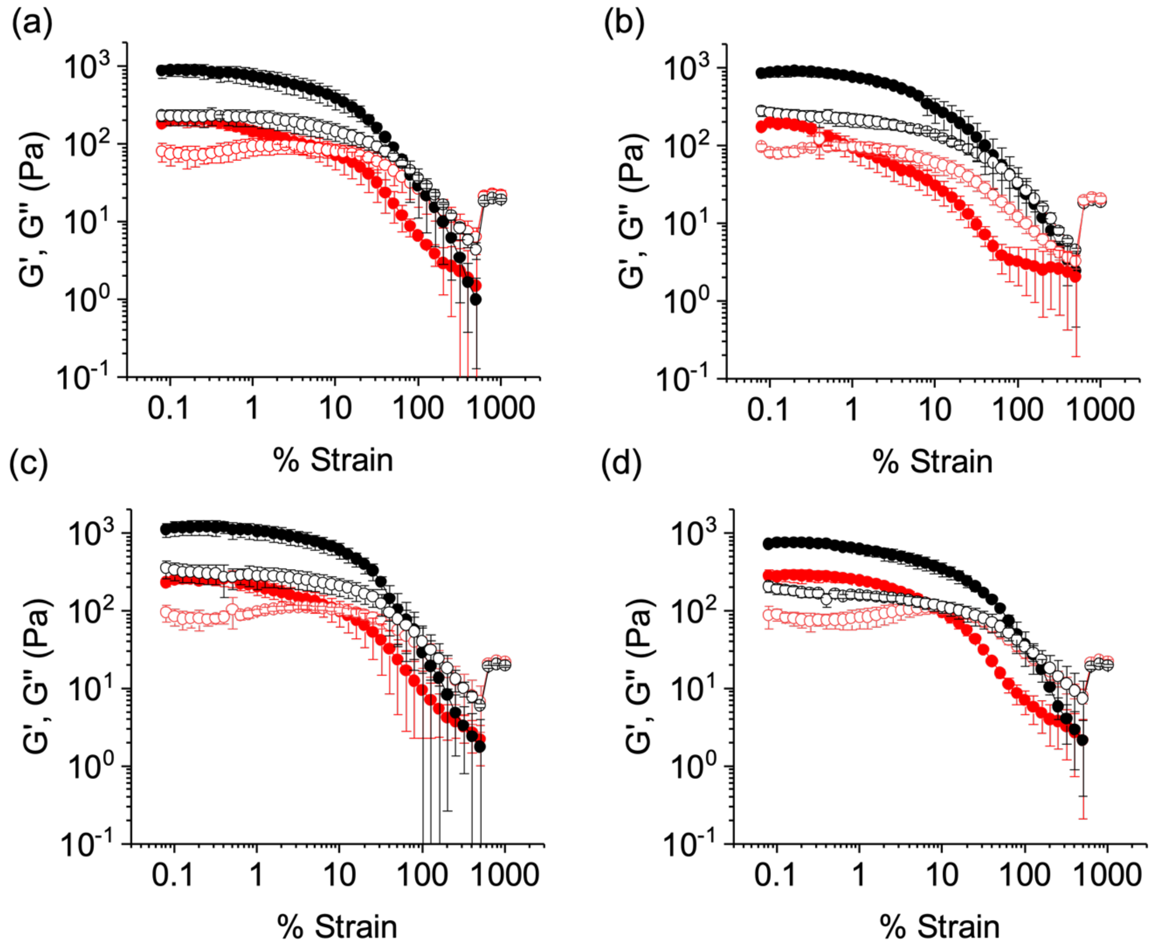

Figure S2. Strain sweeps for gels formed from **CD-005** with (black data) and without (red data) the addition of $CaCl_2$ with (a) 0 wt% added dextran; (b) 0.1 wt% added dextran; (c) 1 wt% added dextran; (d) 10 wt% added dextran. In all cases, the full symbols represent G′ and the open symbols represent G″. Measurements were performed in triplicates and plotted data are presented as mean values ± standard deviation.

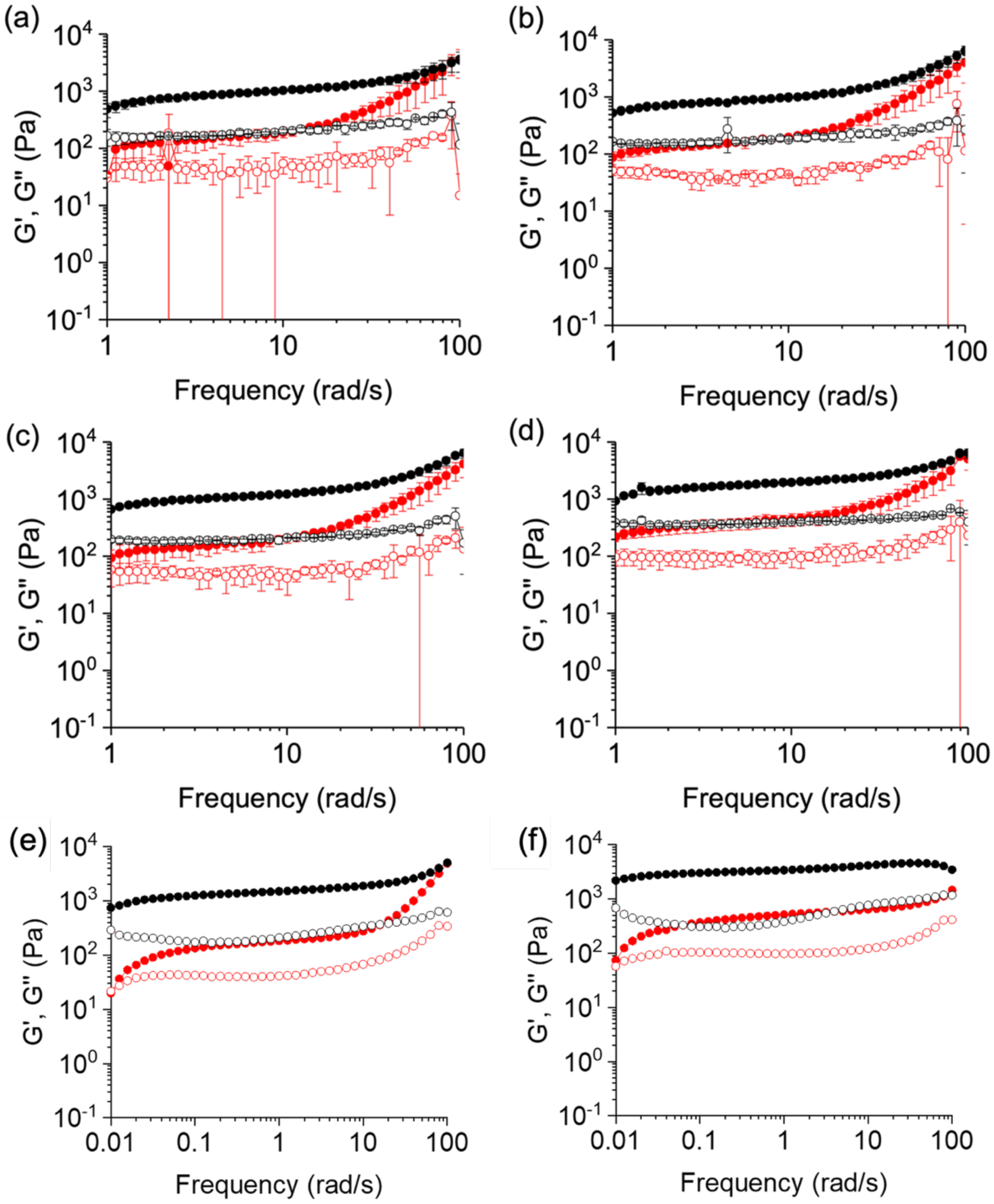

Figure S3. Frequency sweeps for gels formed from **CD-005** with (black data) and without (red data) the addition of $CaCl_2$ with (a) 0 wt% added dextran; (b) 0.1 wt% added dextran; (c) 1 wt% added dextran; (d) 10 wt% added dextran; (e) Frequency sweeps starting at 0.01 rad/s for gels formed from **CD-005** with (black data) and without (red data) the addition of $CaCl_2$ in presence of 0 wt% dextran; (f) Frequency sweeps starting at 0.01 rad/s for gels formed from **CD-005** with (black data) and without (red data) the addition of $CaCl_2$ in presence of 10 wt% dextran. In all cases, the full symbols represent G′ and the open symbols represent G″. Measurements were performed in triplicates and plotted data are presented as mean values ± standard deviation. Note the occasional large error bar is due to the averaging of frequency sweeps where some anomalous data points are not uncommon.

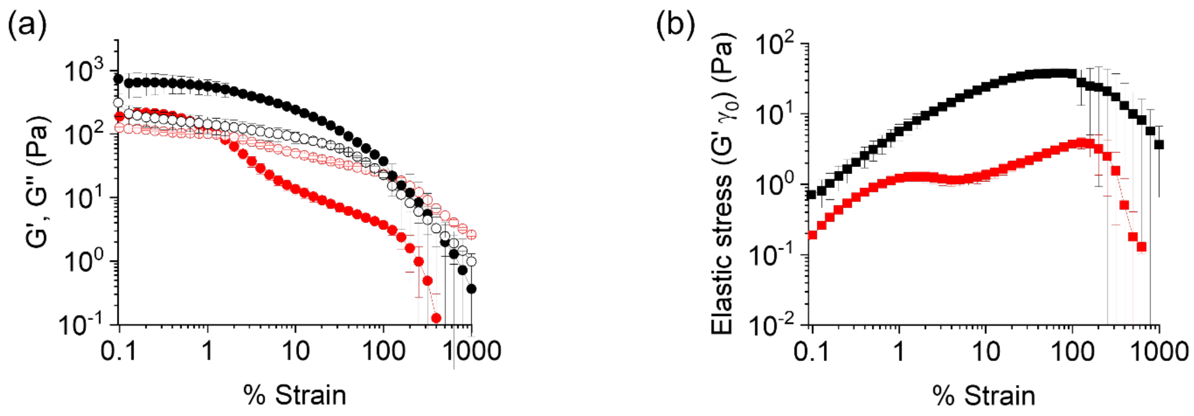

Figure S4. Determination of dynamic yield stress. (a) Amplitude sweeps for gels formed from **CD-005** with (black data) and without (red data) addition of $CaCl_2$ at 1 rad/s. (b) Dynamic yield stress plot for gels formed from **CD-005** with (black data) and without (red data) addition of $CaCl_2$ using the data from (a), as previously used for other systems.[1-5] Here, both samples show an initial linear increase, due to the elastic response of the gel network. For the gels prepared in presence of salt, a single yielding process at a strain amplitude of 63% (38 Pa) can be observed, which agrees with the crossover point of G′ and G″. The gels with no calcium instead show two maxima in the elastic stress curve, with the first occurring at a strain of 1.6% (1.4 Pa) and the second one at 126% (4.06 Pa). The first yield point occurs within the values of the crossover strain, while the second one can be related to the peak of G″, the maxima of energy dissipation. In this case, the first yield might be ascribed to initial breaking of the self-assembled network into smaller disconnected clusters, which are further disrupted at the second yield stress.[3] Measurements were performed in duplicates and plotted data are presented as mean values ± standard deviation.

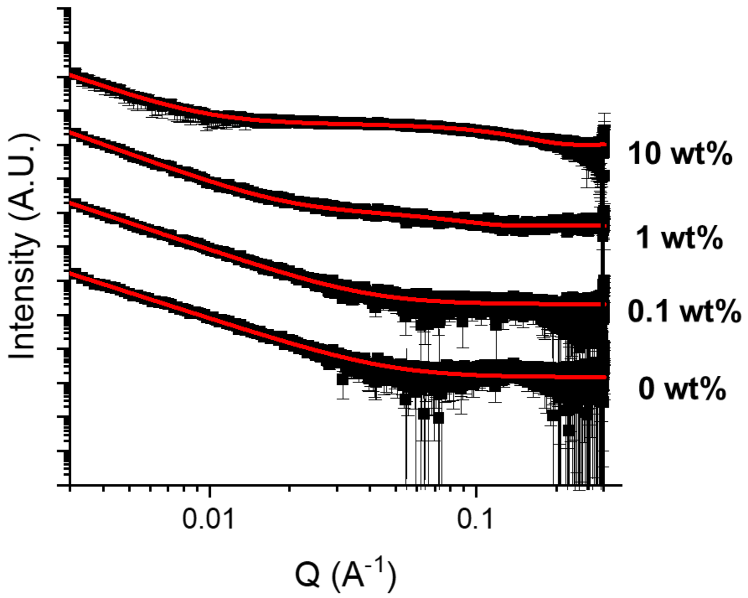

Figure S5. SAXS data for gels formed from **CD-005** with no added $CaCl_2$ containing different wt% of dextran. In all cases, the data are shown as full circles with the fits to the data shown as red lines. The models used to generate the fits and the parameters from the fitting are shown in Table S1. The plotted data show the averaged scattering pattern obtained from five measurements across the sample. The error bars are generated during data processing by calculating scattering signal uncertainty in the detected data according to previously published methods.[6,7]

CD-005 gels, 0 eq. CaCl2

**Power law model**	0 wt% dextran		0.1 wt% dextran		**Power law + Sphere**	1 wt% dextran		10 wt% dextran	
	Value	Error	Value	Error		Value	Error	Value	Error
**Scale**	4.30e-06	1.63e-07	4.51e-06	1.37e-07	**Scale**	1		1	
**Background (cm^{-1})**	0.02		0.02		**Background (cm^{-1})**	0.041		0.099	
**Power**	2.60	0.007	2.63	0.006	**A_scale**	2.17e-04	4.55e-06	4.92e-03	1.43e-05
					**A_radius (Å)**	29.195	0.29	17.64	0.02
					**B_scale**	1.6737e-06	7.51e-08	1.231e-06	9.5e-08
					**B_power**	2.83	8.55×10^{-3}	2.75	0.015
χ^2	2.28		2.04		χ^2	2.07		4.74	

Table S1. Fits to SAXS data for gels formed from **CD-005** with no added $CaCl_2$ containing different wt% of dextran. The data was fit using the Levenberg-Marquardt algorithm in the SasView software (version 5.0.4), with 200 steps and f(x) and x tolerance of 1.5 x 10^{-8}. The errors presented in the table correspond to fitting errors.

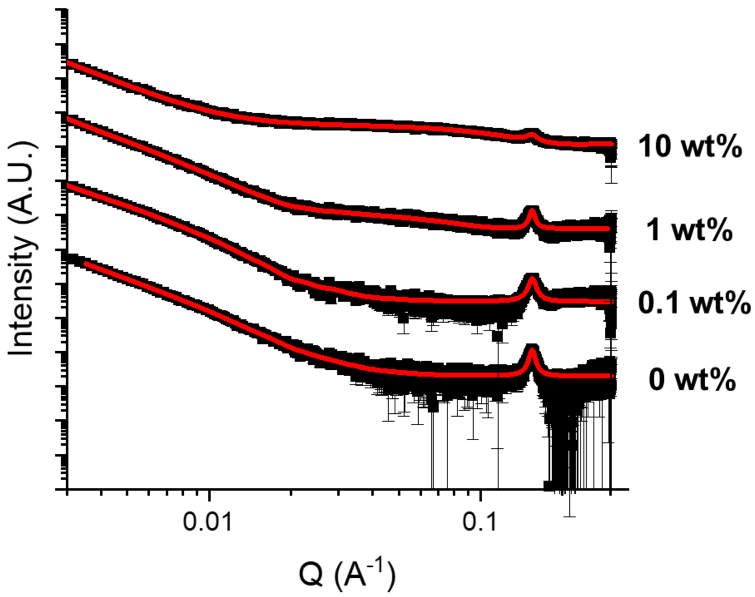

Figure S6. SAXS data for gels formed from **CD-005** with added $CaCl_2$ containing different wt% of dextran. In all cases, the data are shown as full circles with the fits to the data shown as red lines. The models used to generate the fits and the parameters from the fitting are shown in Table S2. The plotted data show the averaged scattering pattern obtained from five measurements across the sample. The error bars are generated during data processing by calculating scattering signal uncertainty in the detected data according to previously published methods.[6,7]

CD-005 gels, 1 eq. CaCl2

**Elliptical cylinder + power law + Lorentzian peak**	0 wt% dextran		0.1 wt% dextran		**Elliptical cylinder + sphere + power law + Lorentzian peak**	1 wt% dextran		**Sphere + power law + Lorentzian peak**	10 wt% dextran	
	Value	Error	Value	Error		Value	Error		Value	Error
**Scale**	1	1	1	1	**Scale**	1		**Scale**	1	
**Background (cm^{-1})**	0.02		0.02		**Background (cm^{-1})**	0.04		**Background (cm^{-1})**	0.11	
**A_scale**	1.78e-05	7.85e-07	4.33e-05	7.15e-07	**A_scale**	2.69e-05	1.28e-08	**A_scale**	0.003	1.75e-05
**A_radius (Å)**	159.29	3.11	165.67	1.18	**A_radius (Å)**	158.6	1.94	**A_radius (Å)**	20.008	0.046
**A_axis_ratio**	2.23	0.09	2.4	0.04	**A_axis_ratio**	2.54	0.07	**B_scale**	4.41e-07	2.07e-08
**A_length (Å)**	1000	/	1000	/	**A_length (Å)**	1000	/	**B_power**	3.09	0.008
**B_scale**	2.36e-07	1.66e-08	1.67e-07	8.56e-09	**B_scale**	0.003	5.35e-05	**C_scale**	0.081	9.41e-04
**B_power**	3.32	0.013	3.45	0.013	**B_radius (Å)**	32.092	0.293	**C_peak_pos**	0.154	
**C_scale**	0.09	0.001	0.11	0.001	**C_scale**	3.49e-08	7.11e-09	**C_peak_HWHM**	0.008	1.54e-04
**C_peak_pos**	0.154	4.96e-05	0.154	4.51e-05	**C_power**	3.65	0.033			
**C_peak_HWHM**	0.004	7.01e-05	0.005	6.36e-05	**D_scale**	0.097	8.38e-04			
					**D_peak_pos**	0.154				
					**D_peak_HWHM**	0.004				
χ^2	2.73		4.63		χ^2	2.20		χ^2	1.74	

Table S2. Fits to SAXS data for gels formed from **CD-005** with added $CaCl_2$ containing different wt% of dextran. The data was fit using the Levenberg-Marquardt algorithm in the SasView software (version 5.0.4), with 200 steps and f(x) and x tolerance of 1.5 x 10^{-8}. The errors presented in the table correspond to fitting errors.

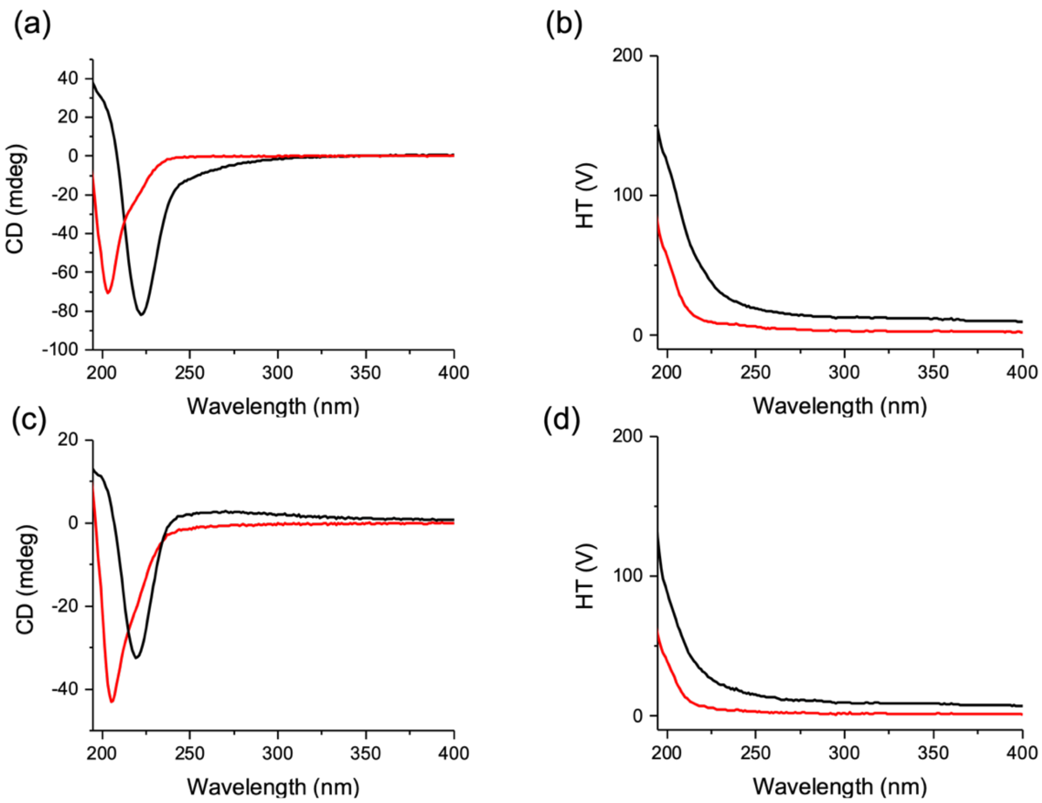

Figure S7. CD and HT data for gels of **CD-005**, in the absence and presence of $CaCl_2$, containing different wt% of dextran. (a) and (b) are for the absence of dextran and (c) and (d) are for gels containing 10wt% dextran. Red data are for the absence of $CaCl_2$ and black data are in the presence of $CaCl_2$. CD was collected in duplicates for each sample and averaged.

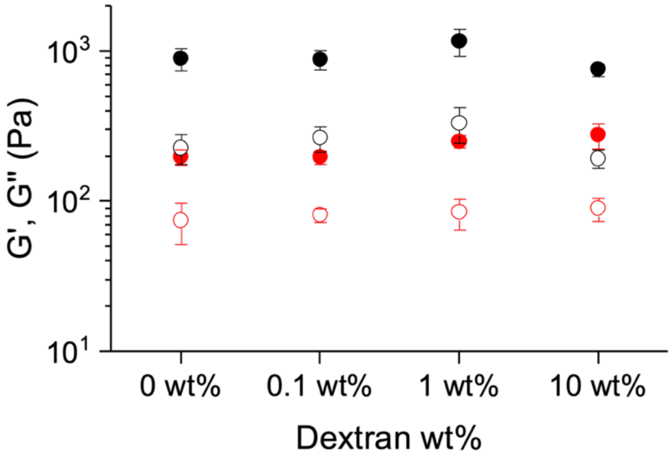

Figure S8. Storage (G′) and loss (G″) moduli for gels formed in the presence of different concentrations of dextran. In all cases, G′ is represented by full circles and G″ by open circles. Red data are for the absence of $CaCl_2$ and black data are in the presence of $CaCl_2$. Measurements were performed in triplicates and plotted data are presented as mean values ± standard deviation.

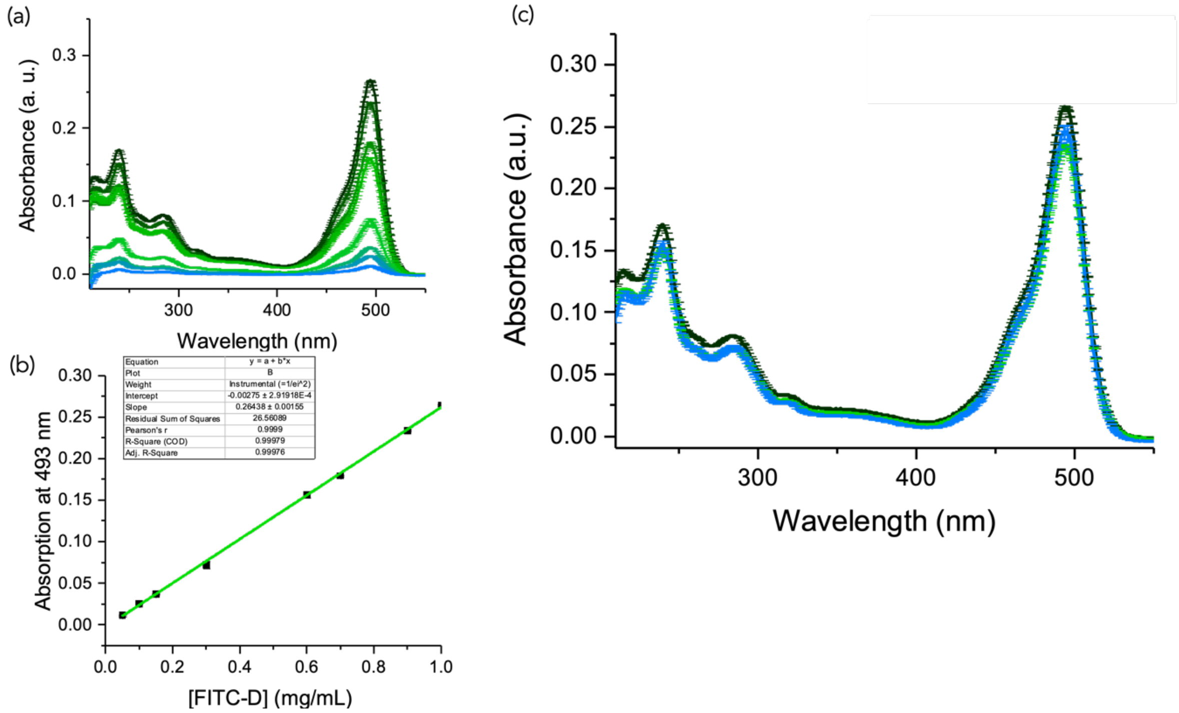

Figure S9. Determination of release of dextran and calibration curves. (a) Spectra of FITC-dextran at different concentrations in buffer used to prepare the calibration curves. (b) Calibration curve for FITC-dextran in buffer. The calibration curve was fit using the Linear fitting function on Origin v2020b. (c) Data for solutions of FITC-dextran at 1 mg/mL (black) and 0.9 mg/mL (green) in buffer along with data from solution extruded from gel (blue) where the solution is expected to be at 1 mg/mL of FITC-dextran. From these data, we release 92 ± 2 % of the expected dextran. All UV-Vis data were collected in triplicates and plotted data are presented as mean values ± standard deviation.

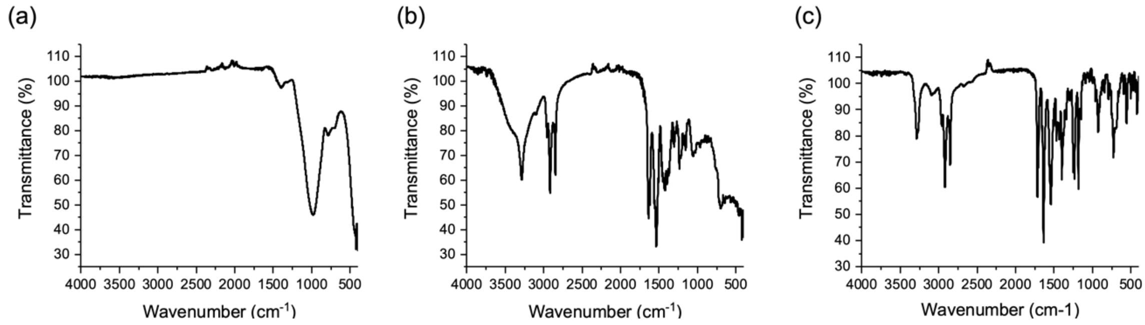

Figure S10. Infrared spectra of (a) clean 2.7 μm syringe filter, (b) 2.7 μm syringe filter after extrusion of **CD-005** gel using the filter, (c) **CD-005** powder as synthesized. Clearly, the syringe filter after extrusion presents peaks coming from the gelator molecule, which are not visible in the spectra of the clean filter.

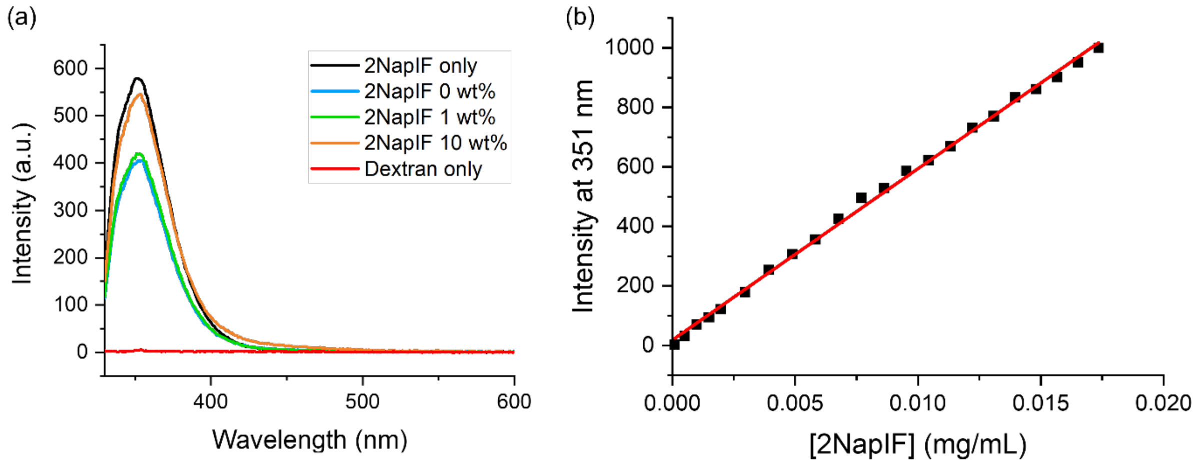

Figure S11. (a) Fluorescence spectra of a solution of 2NapIF (see Figure S1 for structure), a solution of dextran only and solutions of dextran at different concentrations recovered by filtration of a gel through a syringe filter, (b) calibration curve obtained with different dilutions of 2NapIF. The calibration curve was fit using the Linear fitting function on Origin v2020b. Comparison with the calibration curve shows that a very small amount of gelator is present in the recovered solutions (0.36%, 0.28%, 0.26% of the initial gelator quantity for 10 wt%, 1 wt% and 0 wt% dextran respectively).

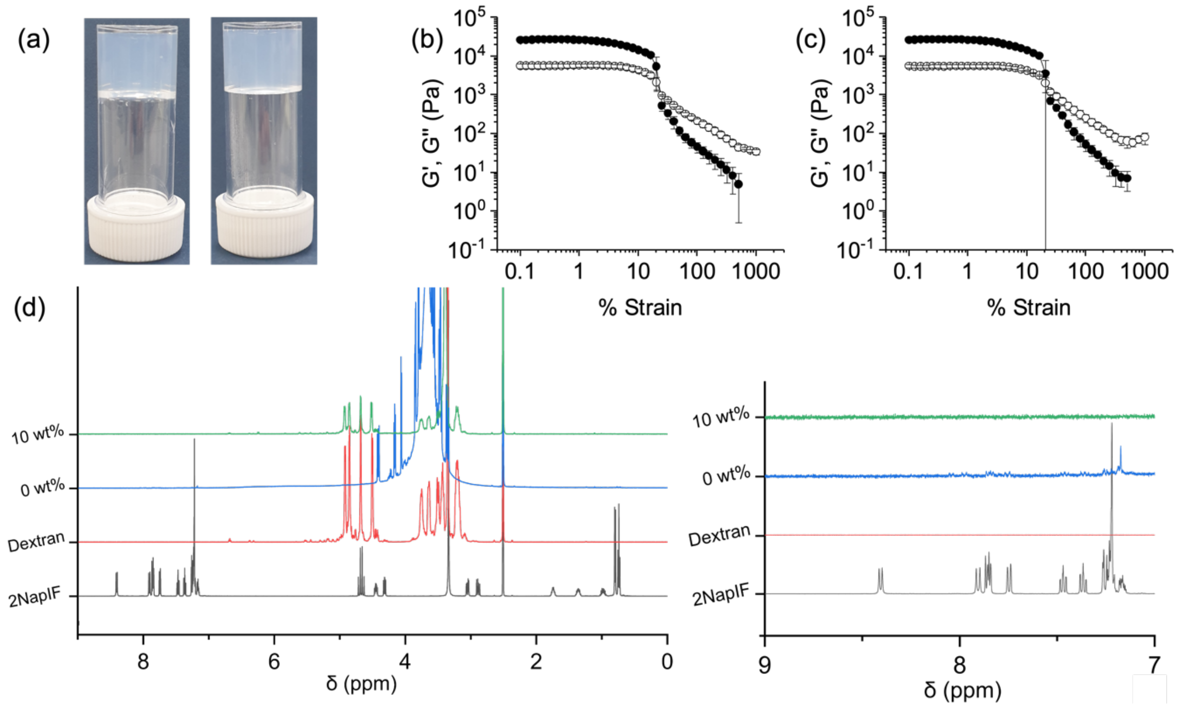

Figure S12. Example data for gels formed using 2NapIF (see Figure S1 for structure) at pH 4. (a) Photographs of gels formed in the absence (left) and presence of 10 wt% dextran (right). (b) Strain sweep for gels formed in the absence of dextran. (c) Strain sweep for gels formed in the presence of 10 wt% dextran. For (b) and (c), the full symbols represent G′ and the open symbols represent G″. Measurements were performed in triplicates and plotted data are presented as mean values ± standard deviation. (d) NMR data for liquid released through the syringe filter showing the presence of the hydrolysis products of GdL (used to change the pH) in both cases and the presence of dextran from the gel containing dextran. The left NMR spectrum shows the full chemical shift range, whilst the right spectrum shows an expansion of the aromatic region to make the absence of gelator clear. The sample collected from the gel formed in the presence of dextran shows the absence of any peaks that can be attributed to the 2NapIF gelator. For the gel formed without dextran, a very small amount of the gelator can be detected on passing through the filter. This can be ascribed to a change in the network on adding dextran.[8]

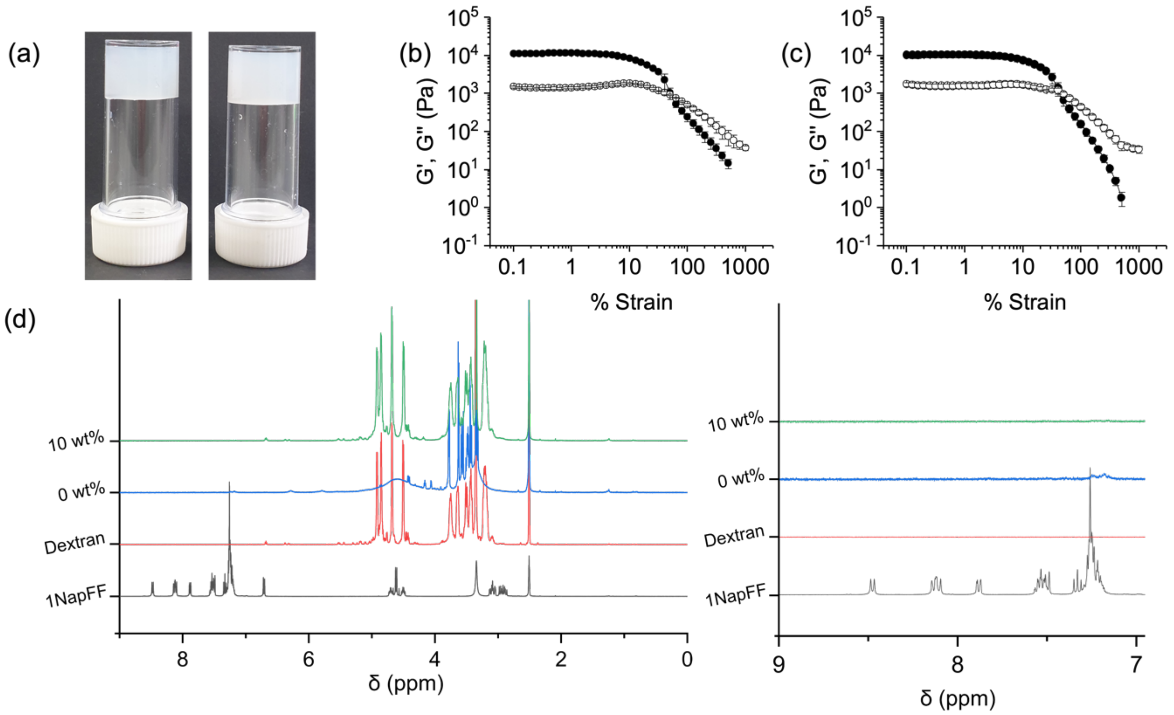

Figure S13. Example data for gels formed using 1NapFF (see Figure S1 for structure) at pH 4. (a) Photographs of gels formed in the absence (left) and presence of 10 wt% dextran (right). (b) Strain sweep for gels formed in the absence of dextran. (c) Strain sweep for gels formed in the presence of 10 wt% dextran. For (b) and (c), the full symbols represent G′ and the open symbols represent G″. Measurements were performed in triplicates and plotted data are presented as mean values ± standard deviation. (d) NMR data for liquid released through the syringe filter showing the presence of the hydrolysis products of GdL (used to change the pH) in both cases and the presence of dextran from the gel containing dextran. The sample collected from the gel formed in the presence of dextran shows the absence of any peaks that can be attributed to the 2NapIF gelator. For the gel formed without dextran, a very small amount of the gelator can be detected on passing through the filter. This can be ascribed to a change in the network on adding dextran.[8]

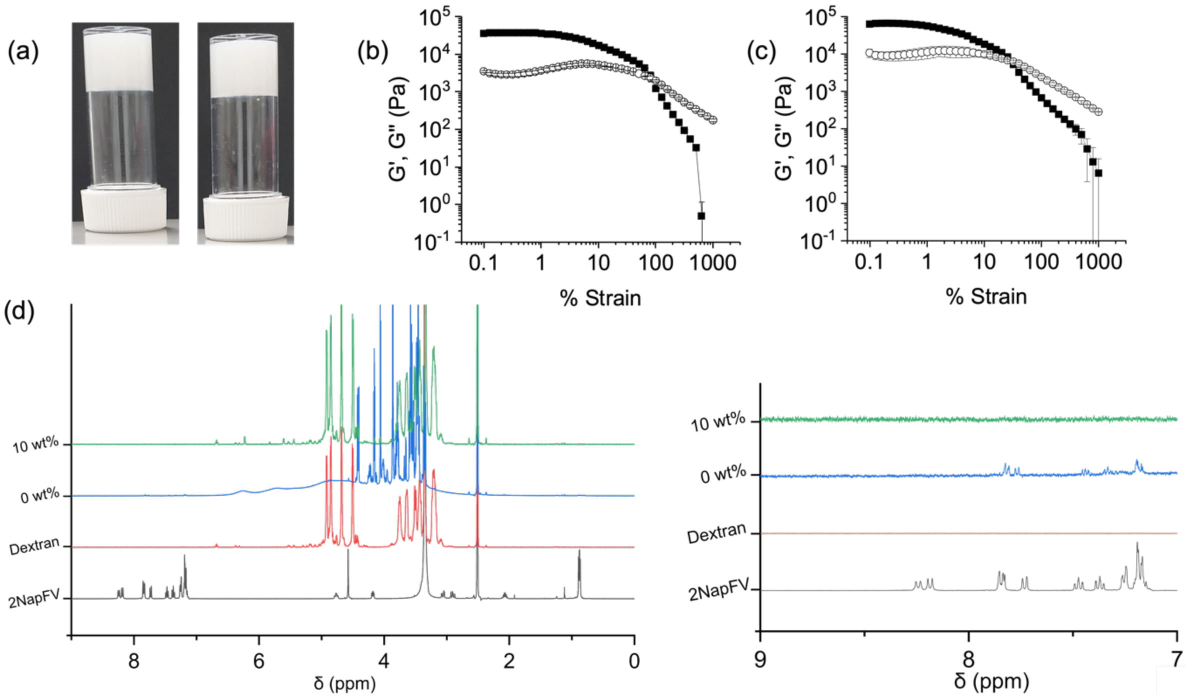

Figure S14. Example data for gels formed using 2NapFV (see Figure S1 for structure) at pH 4. (a) Photographs of gels formed in the absence (left) and presence of 10 wt% dextran (right). (b) Strain sweep for gels formed in the absence of dextran. (c) Strain sweep for gels formed in the presence of 10 wt% dextran. For (b) and (c), the full symbols represent G′ and the open symbols represent G″. Measurements were performed in triplicates and plotted data are presented as mean values ± standard deviation. (d) NMR data for liquid released through the syringe filter showing the presence of the hydrolysis products of GdL (used to change the pH) in both cases and the presence of dextran from the gel containing dextran. The sample collected from the gel formed in the presence of dextran shows the absence of any peaks that can be attributed to the 2NapIF gelator. For the gel formed without dextran, a very small amount of the gelator can be detected on passing through the filter. This can be ascribed to a change in the network on adding dextran.[8]

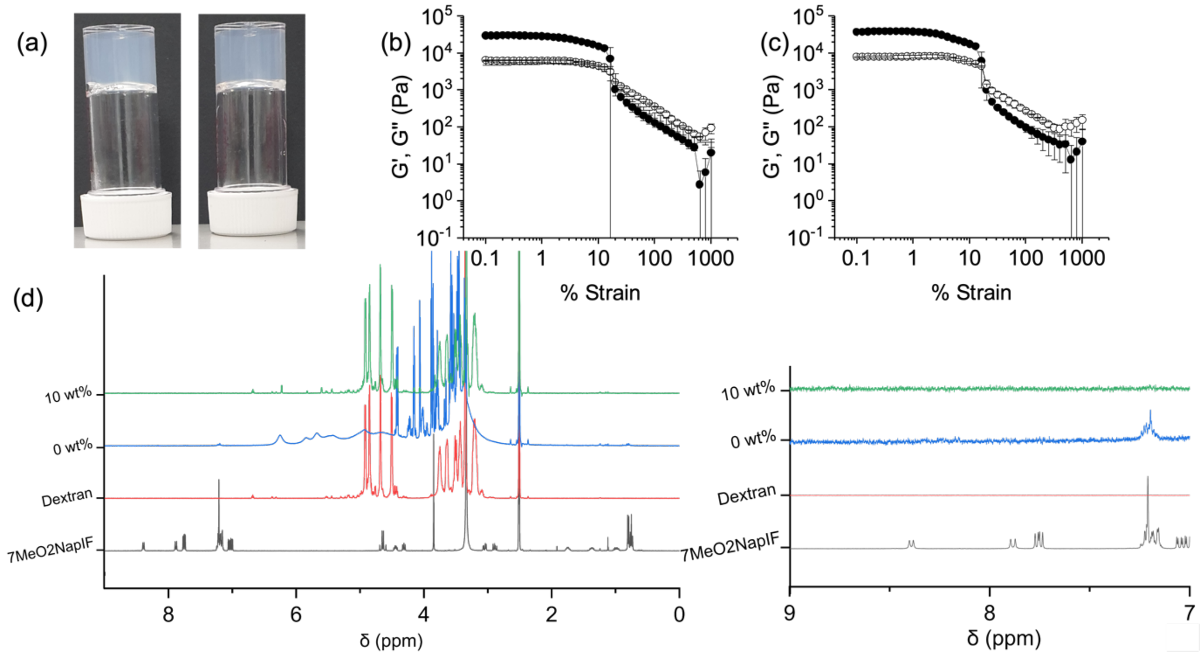

Figure S15. Example data for gels formed using 7MeONap2IF (see Figure S1 for structure) at pH 4. (a) Photographs of gels formed in the absence (left) and presence of 10 wt% dextran (right). (b) Strain sweep for gels formed in the absence of dextran. (c) Strain sweep for gels formed in the presence of 10 wt% dextran. For (b) and (c), the full symbols represent G′ and the open symbols represent G″. Measurements were performed in triplicates and plotted data are presented as mean values ± standard deviation. (d) NMR data for liquid released through the syringe filter showing the presence of the hydrolysis products of GdL (used to change the pH) in both cases and the presence of dextran from the gel containing dextran. The sample collected from the gel formed in the presence of dextran shows the absence of any peaks that can be attributed to the 2NapIF gelator. For the gel formed without dextran, a very small amount of the gelator can be detected on passing through the filter. This can be ascribed to a change in the network on adding dextran.[8]

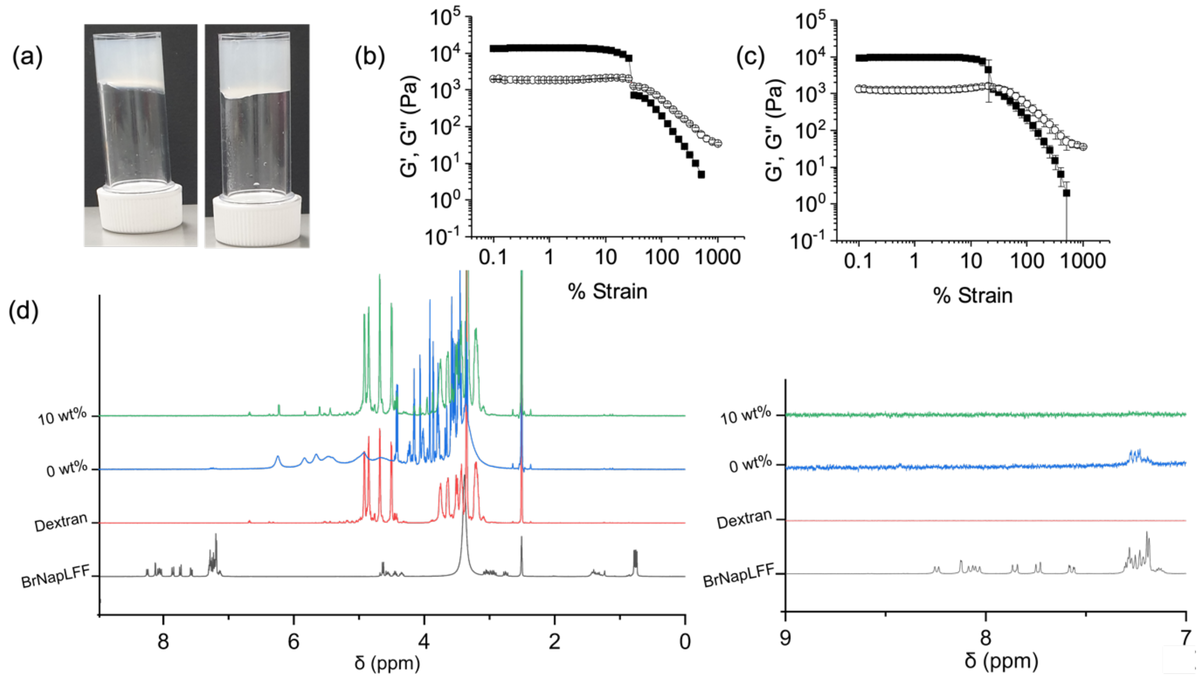

Figure S16. Example data for gels formed using 1Br2NapLFF (see Figure S1 for structure) at pH 4. (a) Photographs of gels formed in the absence (left) and presence of 10 wt% dextran (right). (b) Strain sweep for gels formed in the absence of dextran. (c) Strain sweep for gels formed in the presence of 10 wt% dextran. For (b) and (c), the full symbols represent G′ and the open symbols represent G″. Measurements were performed in triplicates and plotted data are presented as mean values ± standard deviation. (d) NMR data for liquid released through the syringe filter showing the presence of the hydrolysis products of GdL (used to change the pH) in both cases and the presence of dextran from the gel containing dextran. The sample collected from the gel formed in the presence of dextran shows the absence of any peaks that can be attributed to the 2NapIF gelator. For the gel formed without dextran, a very small amount of the gelator can be detected on passing through the filter. This can be ascribed to a change in the network on adding dextran.[8]

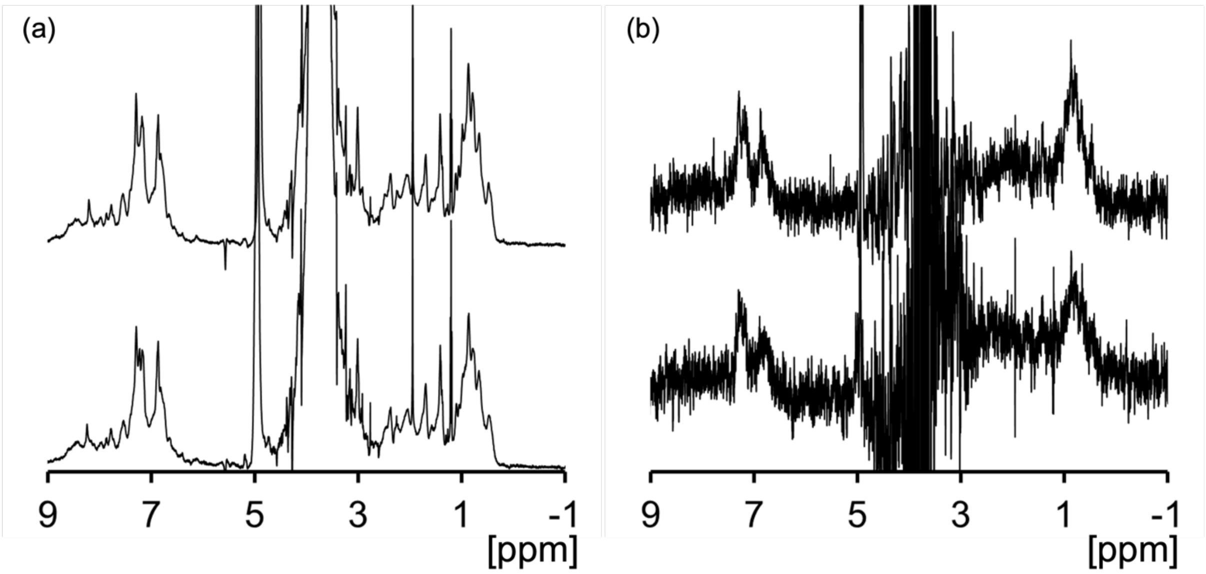

Figure S17. (a) ^{1}H NMR spectrum with perfect echo solvent suppression for (bottom) insulin in buffer and (top) insulin in a **CD-005** gel; in both cases, [insulin] = 3.2 mg/mL. The signals in both cases are very similar in terms of strength and width, strongly implying that the insulin is not absorbed on to the fibers in the gel. (b) STD-NMR with perfect echo solvent suppression for (bottom) insulin in buffer and (top) insulin in a **CD-005** gel; in both cases, [insulin] = 3.2 mg/mL. Presaturation is applied at ≈3.8 ppm.[9] the size of the STD difference peaks are very similar with and without gel, again implying that the insulin is not sticking to the gel fibres.

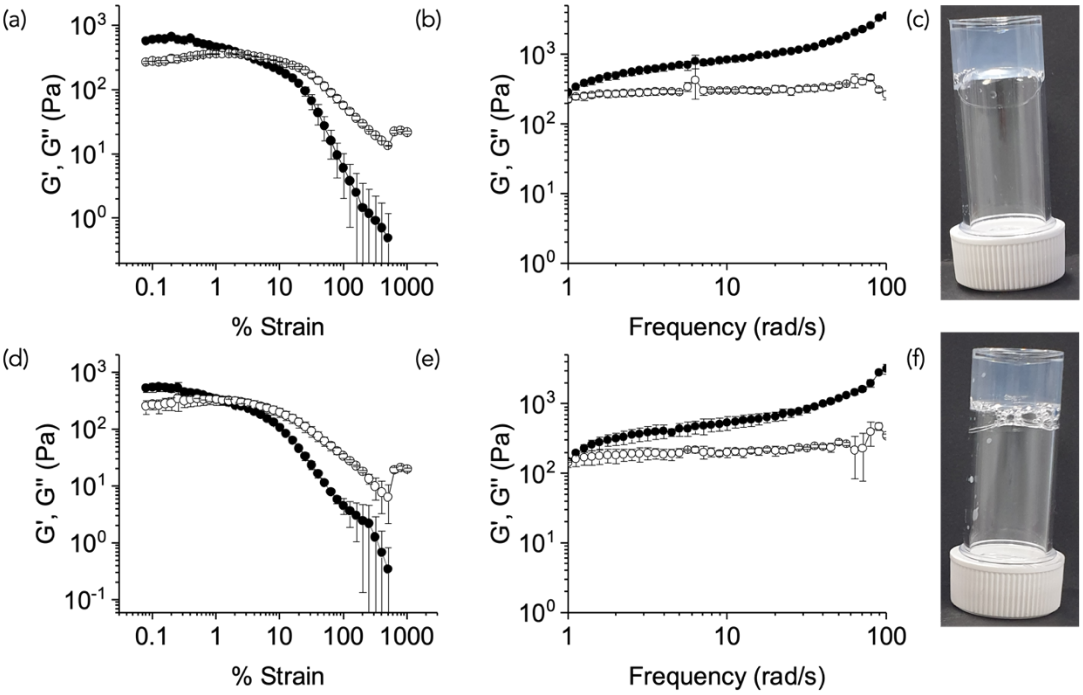

Figure S18. Rheology for gels containing insulin. (a) Strain sweeps for gels formed from **CD-005** with 0.2 mg/mL insulin. (b) Frequency sweeps for gels formed from **CD-005** with 0.2 mg/mL insulin. (c) Photograph of exemplar gel formed in the presence of 0.2 mg/mL insulin. (d) Strain sweeps for gels formed from **CD-005** with 3.2 mg/mL insulin. (e) Frequency sweeps for gels formed from **CD-005** with 3.2 mg/mL insulin. (f) Photograph of exemplar gel formed in the presence of 3.2 mg/mL insulin. For all rheological measurements, data were performed in triplicates and plotted data are presented as mean values ± standard deviation.

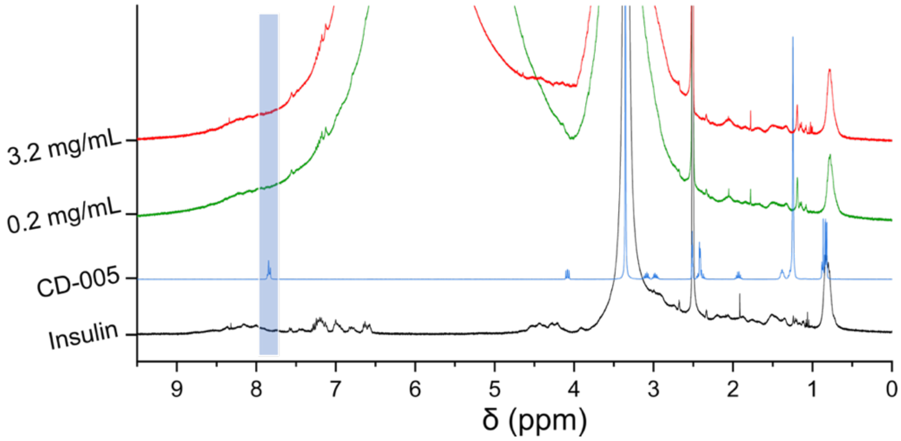

Figure S19. ^{1}H NMR spectra for (bottom to top) insulin, **CD-005**, the liquid released through the syringe filter for a gel containing 0.2 mg/mL insulin and the liquid released through the syringe filter for a gel containing 3.2 mg/mL insulin. The overlaid light blue box shows the peak used to show the absence of the **CD-005** in the extruded samples.

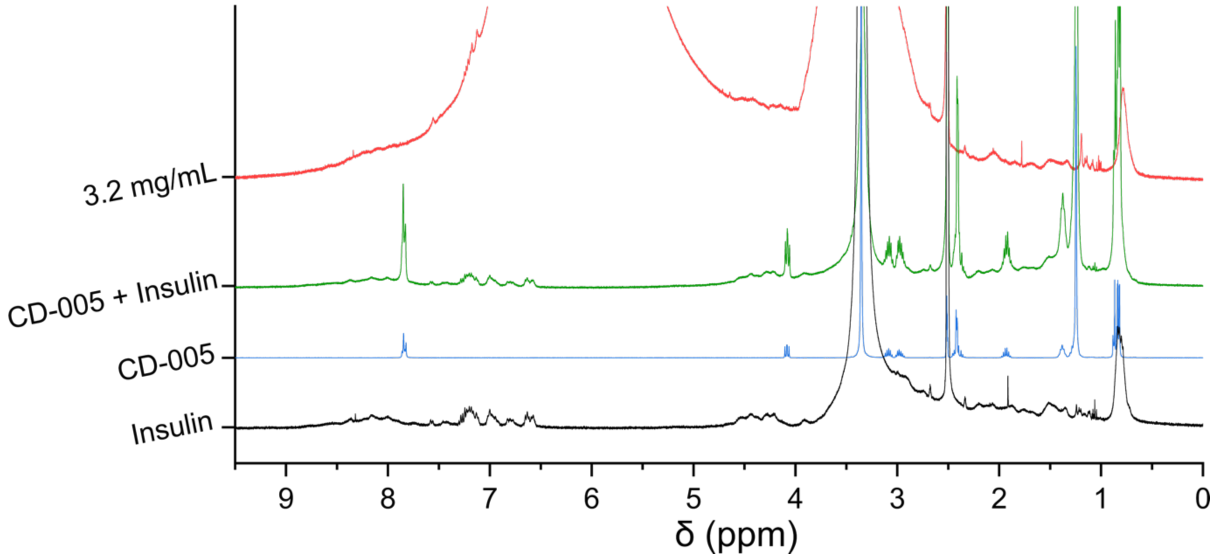

Figure S20. ^{1}H NMR spectra for (bottom to top) insulin, **CD-005**, a mixture of **CD-005** and insulin in the ratios used in the gel (i.e. what would be expected it all gelator were extruded through the filter) and the liquid released through the syringe filter for a gel containing 3.2 mg/mL insulin.

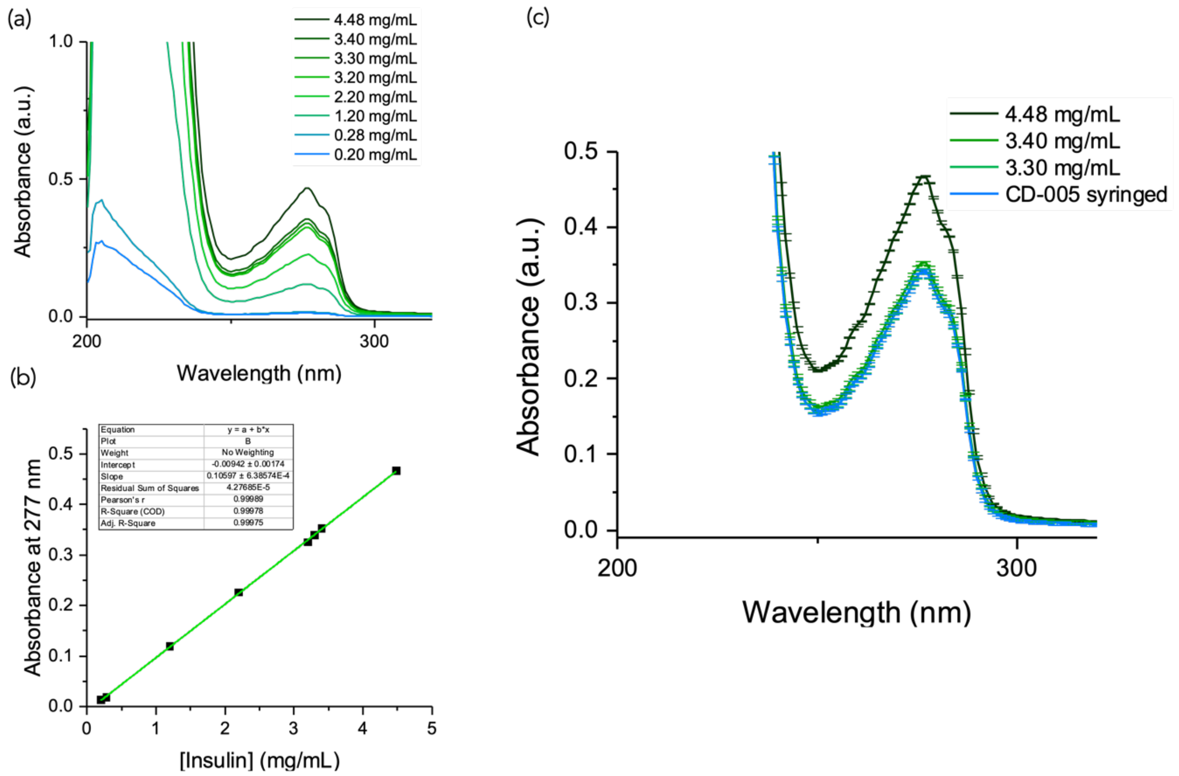

Figure S21. Determination of release of insulin and calibration curves. (a) Spectra of insulin at different concentrations in buffer used to prepare the calibration curves. (b) Calibration curve for insulin in buffer. The calibration curve was fit using the Linear fitting function on Origin v2020b. (c) Data for solutions of insulin at 4.48 mg/mL (black; from calibration curve for comparison), 3.40 mg/mL (dark green; from calibration curve for comparison), 3.30 mg/mL (light green; from calibration curve for comparison) and solution extruded from gel (blue) where the solution is expected to be at 3.2 mg/mL of insulin. From these data, we release 100 ± 1 % of the expected dextran. Data were collected in triplicates and plotted data are presented as mean values ± standard deviation.

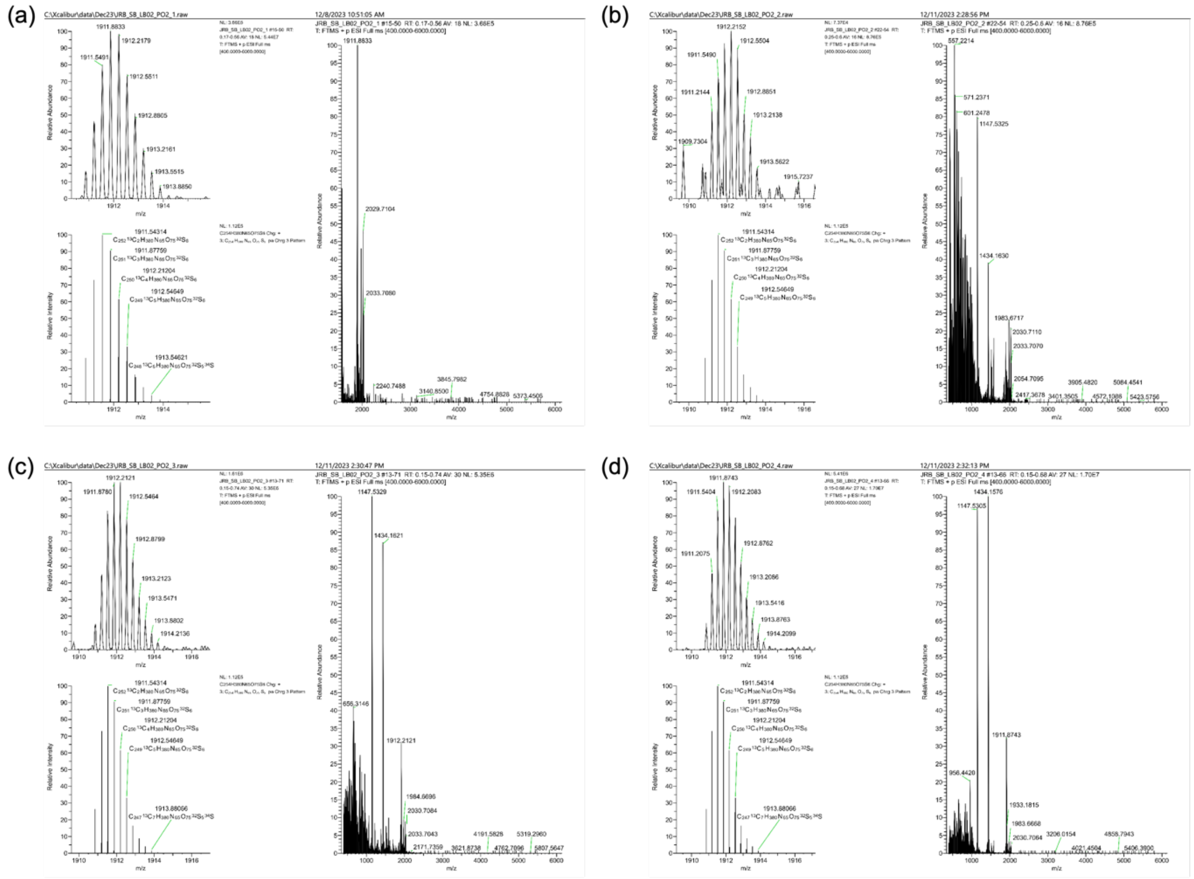

Figure S22. HRMS data. (a) Solution of insulin at a concentration of 0.2 mg/mL; (b) Solution of insulin at a concentration of 0.2 mg/mL collected after extrusion from a gel of **CD-005** through a syringe; (a) Solution of insulin at a concentration of 3.2 mg/mL; (b) Solution of insulin at a concentration of 3.2 mg/mL collected after extrusion from a gel of **CD-005** through a syringe.

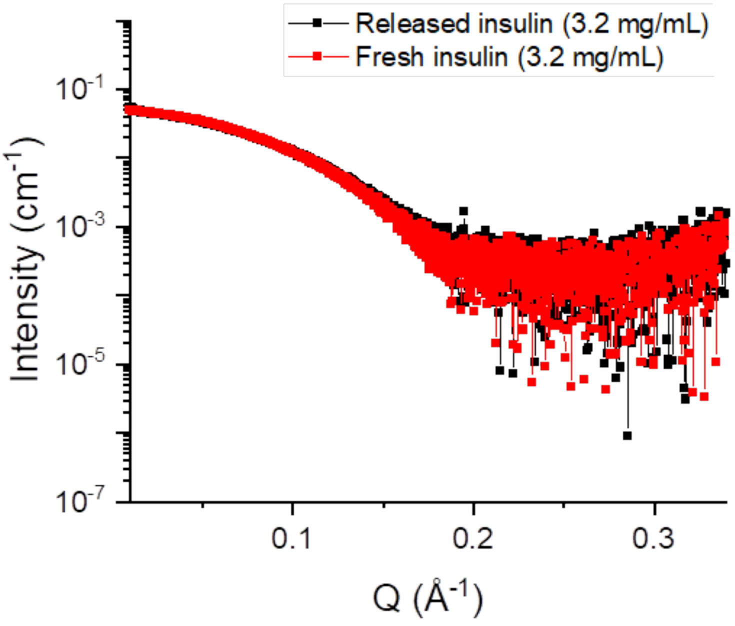

Figure S23. SAXS scattering pattern of fresh insulin (red data) and insulin after being released from the gel (black data). In both cases, the concentration of insulin was 3.2 mg/mL. The plotted data show the averaged scattering pattern obtained from thirty measurements across the sample. The error bars are generated during data processing by calculating scattering signal uncertainty in the detected data according to previously published methods.[6,7]

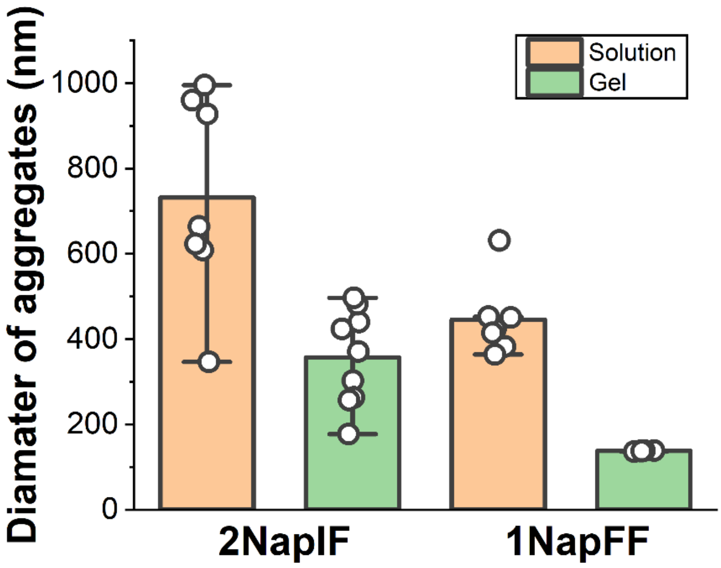

Figure S24. Insulin aggregation assay using dynamic light scattering with alternative gelators (2NapIF in solution, n = 7; 2NapIF in gel, n = 9; 1NapFF in solution, n = 7; 1NapFF in gel, n = 6). Error bars, mean ± s.d. Gels were incubated at 25°C for 24 hours at 900 rpm. The structures of the gelators are shown in Figure S1.

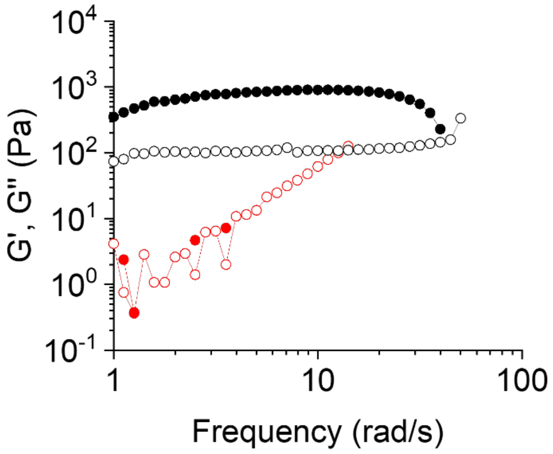

Figure S25. Frequency sweeps for materials formed from **CD-005** with (black data) and without (red data) the addition of $CaCl_2$ at 60°C. Gels were pre-formed in cups and then heated on the rheometer in the cups. In all cases, the full symbols represent G′ and the open symbols represent G″. At this temperature, the materials without $CaCl_2$ are not gels, shown by G″ > G′ (no values are plotted in many cases and the values are <0 and we are plotting on a log scale). The materials with $CaCl_2$ have more gel-like properties are this temperature, but still show that G′ and G″ cross-over at higher frequency (likely due to inertial effects in the rheometer). Nonetheless, these materials are solid-like to the eye at this temperature and the sample can be inverted without flow.

DATA REPORT

Alarm ☒ ID: TZ2302038979

File Information

File Created Date:	05/26/23 14:02:36

Note: All Times shown are based on UTC+0:00 and 24-Hour clock [MM/DD/YY HH:MM:SS]

Device Information

Device Type:	TempU05	Firmware Version:	5.20
ID:	TZ2302038979		
Start Delay:	30 min	Log Interval:	10 min
Description:	Temperature Recording		

Logging Summary

First Point:	05/22/23 12:45:41	Max:	24.2°C
Stop Time:	05/25/23 13:05:41	Min:	17.6°C
Number of Points:	435	MKT:	21.1°C
Trip Length:	03d 00h 20m 00s	Average:	21.1°C
Stop Mode:	Manual Stop		

Alarm Condition	First Point Time	Time of Violations	No. of Violations	Status
Over 8.0°C	05/22/23 12:45:41	03d 00h 20m 00s	435	Alarm
Below 2.0°C	00/00/00 00:00:00	00d 00h 00m 00s	0	OK

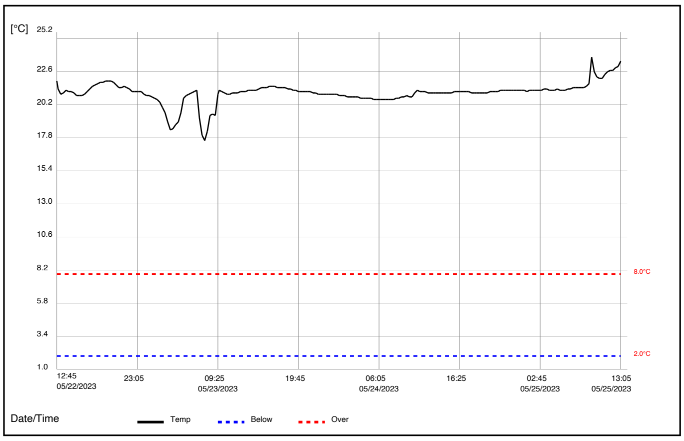

Figure S26. Temperature log for posted gel samples.

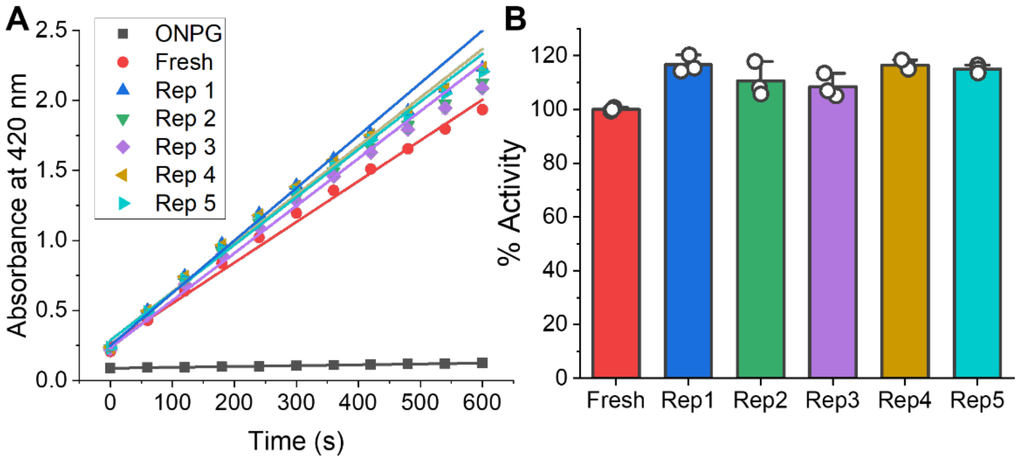

Figure S27. Recovery of beta-galactoside from **CD-005** gels following return postage (n = 3). A) Raw data of ONPG hydrolysis by beta-galactosidase; B) Activity recovery as a percentage of fresh (controlled to total protein concentration). Error bars, mean ± s.d.

Synthesis and characterisation of CD-005

tert-Butyl *N*-[(1*S*)-1-(dodecylcarbamoyl)-2-methylpropyl]carbamate (**CD-001**)

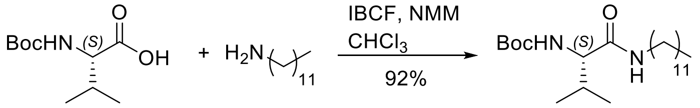

Boc-Val-OH **CD-001**

To a solution of Boc-Val-OH (2.71 g, 12.5 mmol) in chloroform (30 mL) cooled in ice/water was added *iso*-butyl chloroformate (1.1 eq, 1.78 mL) followed by *N*-methylmorpholine (1.15 eq, 1.58 mL). The mixture was stirred for 45 minutes and developed a precipitate during this time. Dodecylamine (1.05 eq, 2.43 g) and another portion of *N*-methylmorpholine (1.15 eq, 1.58 mL) were added and the reaction was allowed to warm to room temperature overnight. After this time, it was diluted with chloroform, washed with water (2x) then brine, dried ($MgSO_4$) and evaporated under reduced pressure. Remaining water was removed by azeotropic evaporation with acetonitrile. The title compound **CD-001** was obtained as a white solid (4.40 g, 92%). Analytical data consistent with structure, not all ^{13}C signals resolved. δ_H (500 MHz, ppm, $CDCl_3$) 5.91 (1H, br s, N$\underline{H}$CH_2), 5.05 (1H, br d, *J* 4.50 Hz, N$\underline{H}$CH*), 3.82 (1H, dd, *J* 8.80, 6.45 Hz, C$\underline{H*}$), 3.30-3.18 (2H, m, C$\underline{H_2}$NH), 2.11 (1H, br d, *J* 5.50, C$\underline{H}$(CH_3)$_2$), 1.52-1.46 (2H, m, C$\underline{H_2}$CH_2NH), 1.43 (9H, s, C(C$\underline{H_3}$)$_3$), 1.31-1.25 (18H, m, all remaining CH_2), 0.95 (3H, d, *J* 6.75, CH(C$\underline{H_3}$)$_2$), 0.91 (3H, d, *J* 6.75, CH(C$\underline{H_3}$)$_2$), 0.88 (3H, t, *J* 6.98 Hz, CH_2C$\underline{H_3}$). δ_C (100 MHz, ppm, $CDCl_3$) 171.66 and 156.08 ($\underline{C}$=O), 79.80 ($\underline{C}$(CH_3)$_3$), 60.30 ($\underline{C}$H*), 39.57 ($\underline{C}H_2$NH), 32.02 ($\underline{C}H_2$), 31.00 ($\underline{C}$H(CH_3)$_2$), 29.75, 29.73, 29.69, 29.64, 29.45 and 29.39 ($\underline{C}H_2$), 28.44 (C($\underline{C}H_3$)$_3$), 27.02 ($\underline{C}H_2$) and 22.79 ($\underline{C}H_2CH_3$), 19.41 and 18.14 (CH($\underline{C}H_3$)$_2$), 14.21 ($CH_2\underline{C}H_3$). HRMS (ESI) m/z: [M+Na]$^+$ calcd for $C_{22}H_{44}N_2NaO_3$ 407.3244; found 407.3228.

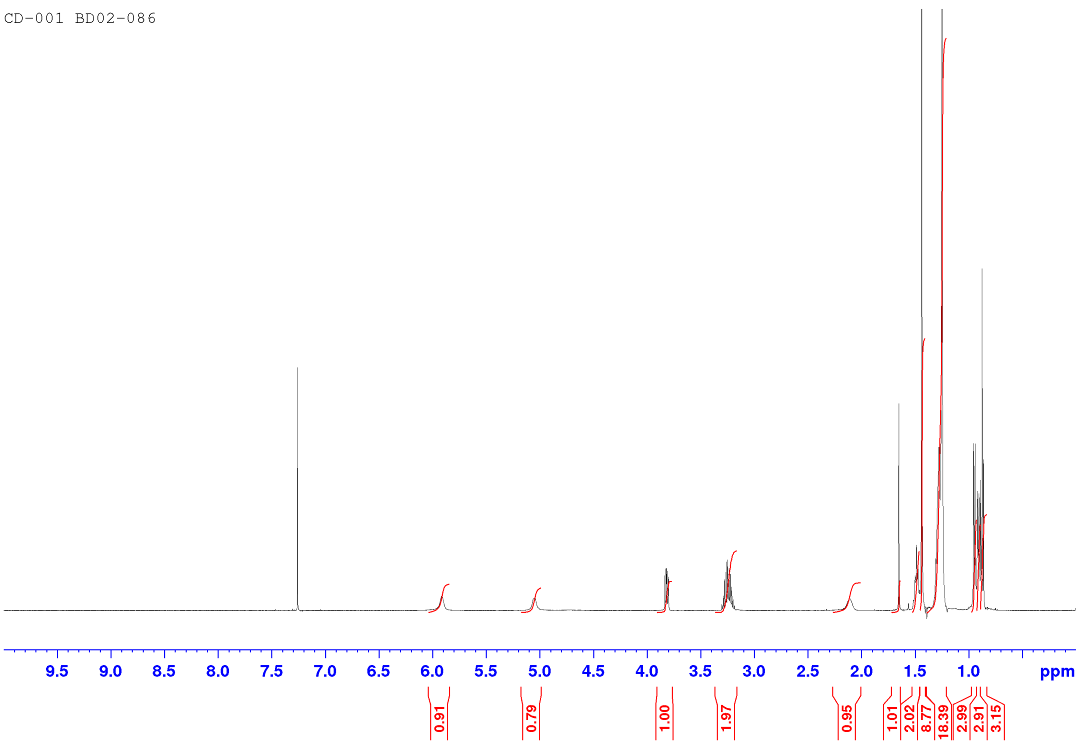

Figure S28. ^{1}H NMR spectrum of **CD-001** in $CDCl_3$.

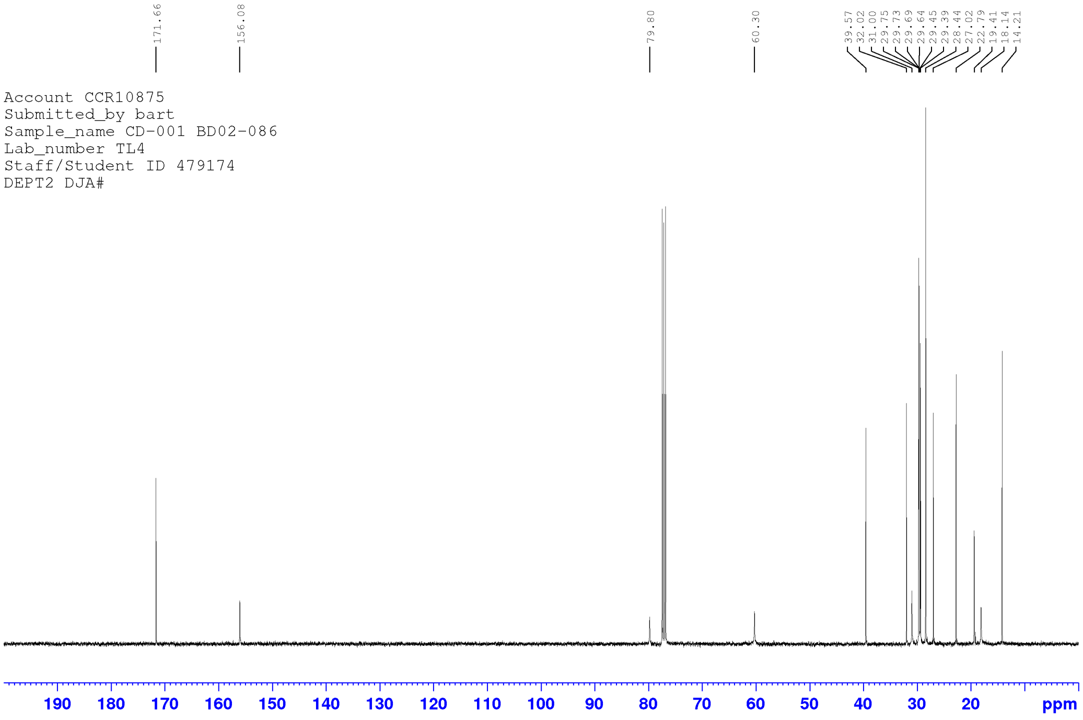

Figure S29. ^{13}C NMR spectrum of **CD-001** in $CDCl_3$.

(2*S*)-2-Amino-*N*-dodecyl-3-methylbutanamide (**CD-003**)

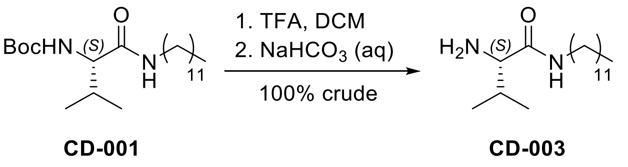

To a solution of **CD-001** (4.34 g, 11.3 mmol) in dichloromethane (50 mL) was added trifluoroacetic acid (*ca.* 30 eq, 25 mL) and the mixture was stirred at ambient temperature overnight. After this time, the reaction was diluted with *iso*-octane and evaporated under reduced pressure. The resulting orange oil was taken up in chloroform, washed in turn with $NaHCO_3$ (aq) (2x) (care: effervescence), and brine, dried ($MgSO_4$), and evaporated to dryness under reduced pressure. The title compound **CD-003** was thus obtained as a pale yellow/brown oil, which solidified on standing in 107% (3.45 g) crude yield and was not purified any further. Analytical data consistent with structure, not all ^{13}C signals resolved. δ_H (500 MHz, ppm, $CDCl_3$) 7.25 (1H, br s, N$\underline{H}$CH$_2$), 3.31-3.17 (3H, m, NHC$\underline{H_2}$ and C$\underline{H}$*), 2.30 (1H, m, C$\underline{H}$(CH$_3$)$_2$), 1.52-1.47 (2H, m, NHCH$_2$C$\underline{H_2}$), 1.42-1.25 (22H, br m, N$\underline{H_2}$ and remaining C$\underline{H_2}$), 0.98 (3H, d, *J* 7.00 Hz, CH(C$\underline{H_3}$)$_2$), 0.87 (3H, t, *J* 6.98 Hz, CH$_2$C$\underline{H_3}$), 0.81 (3H, d, *J* 6.85 Hz, CH(C$\underline{H_3}$)$_2$). δ_C (100 MHz, ppm, $CDCl_3$) 174.27 ($\underline{C}$=O), 60.28 ($\underline{C}$H*), 39.07 (NH$\underline{C}$H$_2$), 31.99 ($\underline{C}$H$_2$), 30.90 ($\underline{C}$H(CH$_3$)$_2$), 29.78, 29.71, 29.66, 29.42, 29.38 and 27.06 ($\underline{C}$H$_2$), 22.76 ($\underline{C}$H$_2$CH$_3$), 19.82 and 16.07 (CH($\underline{C}$H$_3$)$_2$), 14.18 (CH$_2\underline{C}$H$_3$). HRMS (ESI) m/z: $[M+Na]^+$ calcd for $C_{17}H_{36}N_2NaO$ 307.2720; found 307.2711.

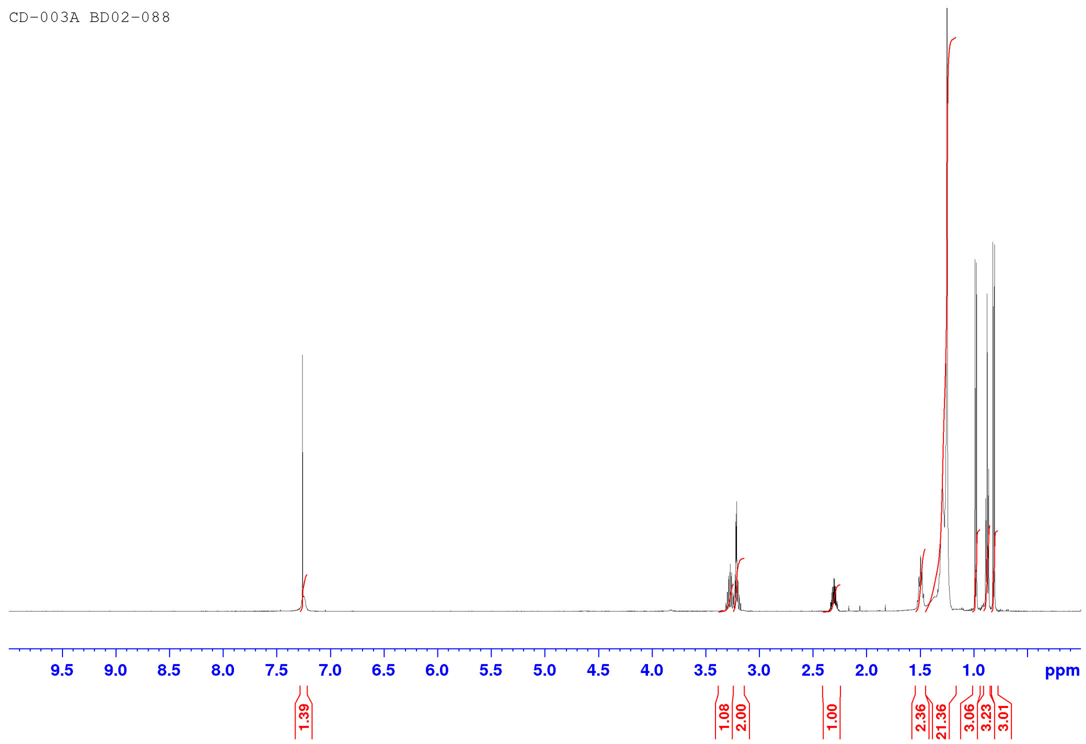

Figure S30. ^{1}H NMR spectrum of **CD-003** in $CDCl_3$.

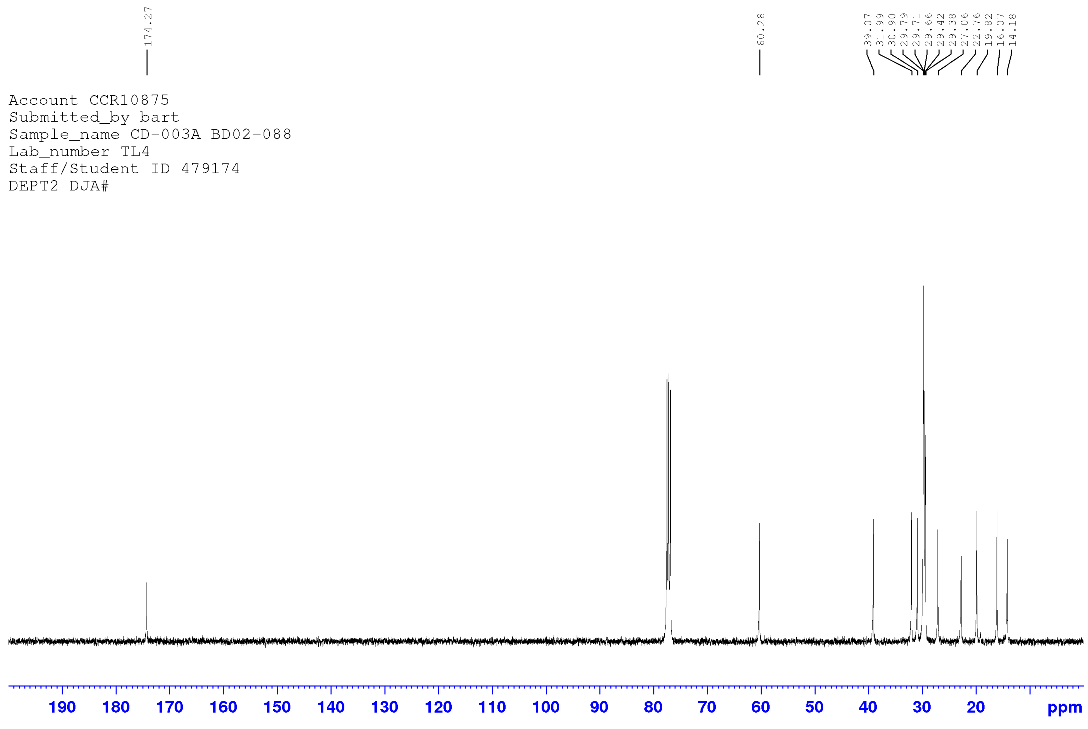

Figure S31. ^{13}C NMR spectrum of **CD-003** in $CDCl_3$.

3-{[(1*S*)-1-(Dodecylcarbamoyl)-2-methylpropyl]carbamoyl}propanoic acid (**CD-005**)

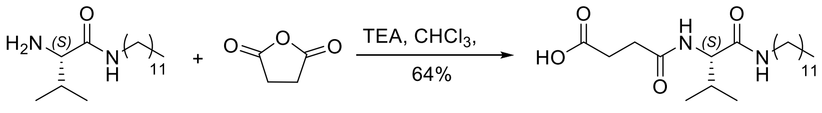

CD-003 **CD-005**

To a solution of **CD-003** (3.10 g, 10.9 mmol) in chloroform (50 mL) were added succinic anhydride (1 eq, 1.09 g) and triethylamine (2.2 eq, 3.34 mL), a reflux condenser was fitted to the flask, and the mixture was heated at 60 °C overnight. After this time, it was diluted with chloroform, washed with 1M HCl (aq) and the layers separated immediately. On standing for *ca.* 20 minutes, the organic phase acquired a gel-like consistency. This was filtered, washed in the filter with additional chloroform and dried under reduced pressure. The resulting solid contained some triethylammonium chloride, as evidenced by NMR. Purification was carried out by recrystallization from boiling acetone, affording the title compound **CD-005** as a white solid (2.67 g, 64%). Analytical data consistent with structure, not all ^{13}C signals resolved. δ_H (500 MHz, ppm, DMSO-d_6) 12.04 (1H, br s, COOH), 7.84-7.81 (2H, m, NH), 4.04 (1H, dd, *J* 8.95, 6.95 Hz, CH*), 3.11-3.05 (1H, m, NHCH_aH_b), 2.99-2.93 (1H, m, NHCH_aH_b), 2.45-2.33 (4H, m, $HO_2C(CH_2)_2$), 1.92 (1H, dq, *J* 6.75, 13.60 Hz, $CH(CH_3)_2$), 1.36 (2H, tt, *J* 9.43, 6.32 Hz, $NHCH_2CH_2$), 1.28-1.17 (18H, m, remaining CH_2), 0.85 (3H, t, *J* 6.93 Hz, CH_2CH_3), 0.82 (3H, d, *J* 2.15, $CH(CH_3)_2$), 0.81 (3H, d, J 2.15, $CH(CH_3)_2$). δ_C (100 MHz, ppm, DMSO-d_6) 173.92, 171.02 and 170.75 (C=O), 57.80 (CH*), 38.36 ($NHCH_2$), 31.33, 30.47, 29.92, 29.28, 29.08, 29.05, 29.03, 29.01, 28.74 and 26.36 (CH_2 and $CH(CH_3)_2$), 22.12 (CH_2CH_3), 19.18 and 18.14 ($CH(CH_3)_2$), 13.94 (CH_2CH_3). HRMS (ESI) m/z: $[M+Na]^+$ calcd for $C_{21}H_{40}N_2NaO_4$ 407.2880; found 407.2878.

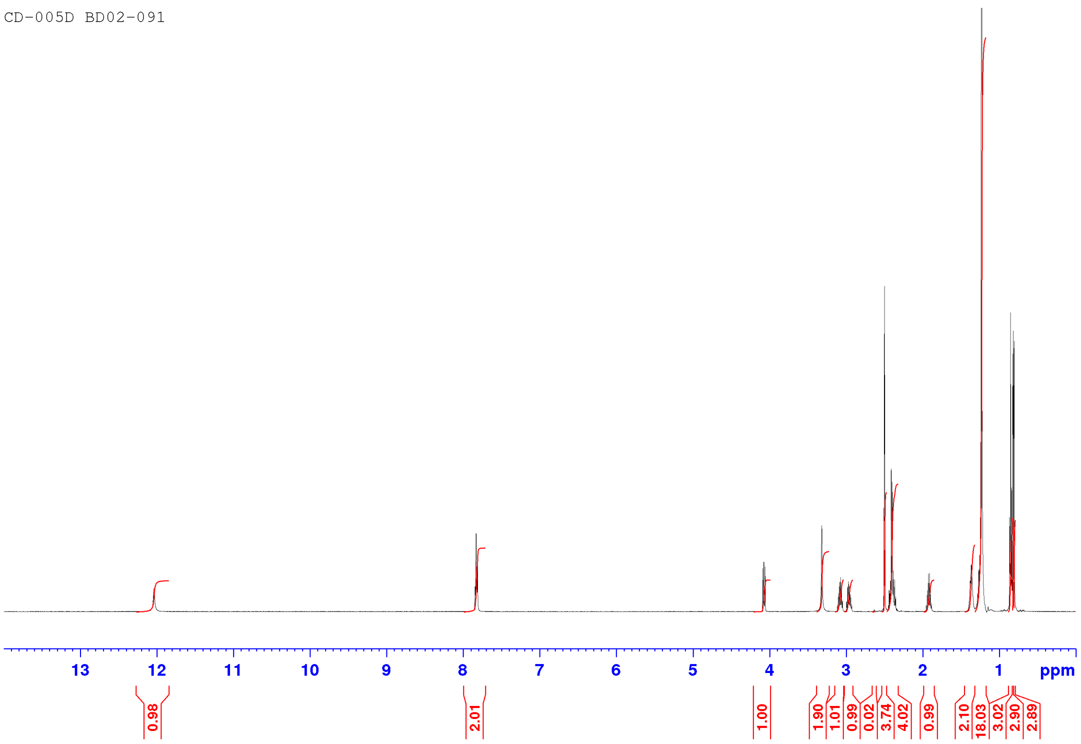

Figure S32. ^{1}H NMR spectrum of **CD-005** in DMSO-d$_6$.

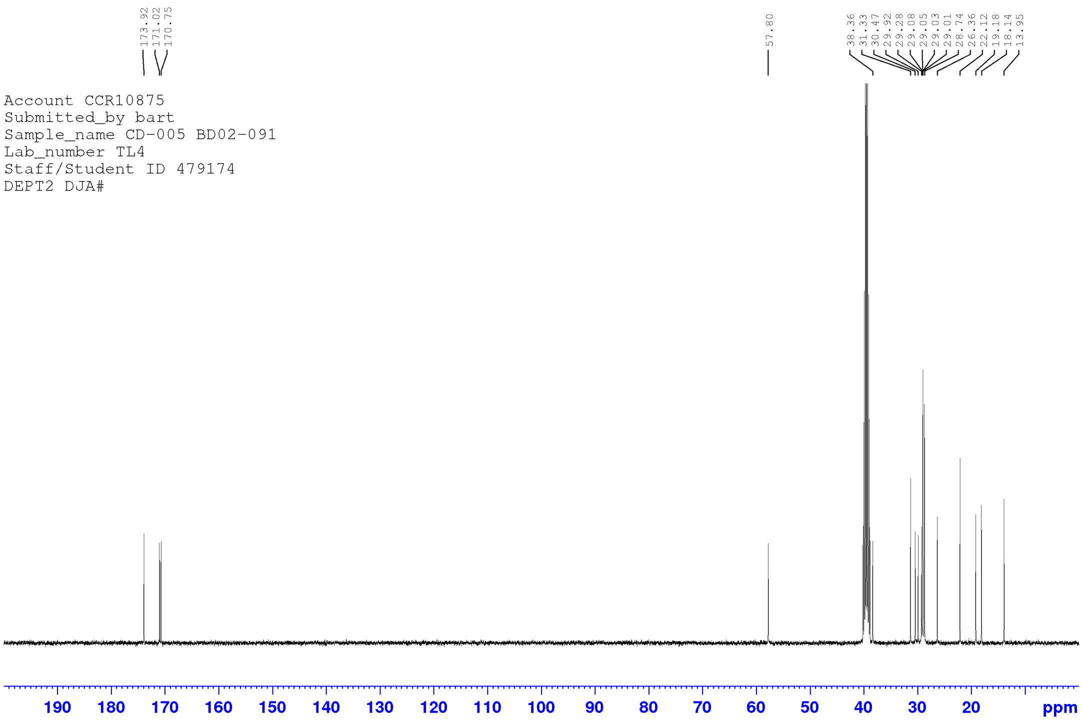

Figure S33. ^{13}C NMR spectrum of **CD-005** in DMSO-d$_6$.

References

1 Yang, M. C., Scriven, L. E. & Macosko, C. W. Some Rheological Measurements on Magnetic Iron Oxide Suspensions in Silicone Oil. *Journal of Rheology* **30**, 1015-1029, doi:10.1122/1.549892 (1986).

2 Walls, H. J., Caines, S. B., Sanchez, A. M. & Khan, S. A. Yield stress and wall slip phenomena in colloidal silica gels. *Journal of Rheology* **47**, 847-868, doi:10.1122/1.1574023 (2003).

3 Laurati, M., Egelhaaf, S. U. & Petekidis, G. Nonlinear rheology of colloidal gels with intermediate volume fraction. *Journal of Rheology* **55**, 673-706, doi:10.1122/1.3571554 (2011).

4 Castro, M., Giles, D. W., Macosko, C. W. & Moaddel, T. Comparison of methods to measure yield stress of soft solids. *Journal of Rheology* **54**, 81-94, doi:10.1122/1.3248001 (2010).

5 Das, M. & Petekidis, G. Shear induced tuning and memory effects in colloidal gels of rods and spheres. *The Journal of Chemical Physics* **157**, doi:10.1063/5.0129709 (2022).

6 Pauw, B. R. Everything SAXS: small-angle scattering pattern collection and correction. *Journal of Physics: Condensed Matter* **25**, 383201, doi:10.1088/0953-8984/25/38/383201 (2013).

7 Pauw, B. R., Smith, A. J., Snow, T., Terrill, N. J. & Thunemann, A. F. The modular small-angle X-ray scattering data correction sequence. *Journal of Applied Crystallography* **50**, 1800-1811, doi:doi:10.1107/S1600576717015096 (2017).

8 Chen, L. *et al.* Low molecular weight gelator–dextran composites. *Chemical Communications* **46**, 6738-6740, doi:10.1039/C0CC01842B (2010).

9 Wallace, M., Iggo, J. A. & Adams, D. J. Probing the surface chemistry of self-assembled peptide hydrogels using solution-state NMR spectroscopy. *Soft Matter* **13**, 1716-1727, doi:10.1039/C6SM02404A (2017).
